# Breakthrough Potential in Near-Infrared Spectroscopy: Spectra Simulation. A Review of Recent Developments

**DOI:** 10.3389/fchem.2019.00048

**Published:** 2019-02-22

**Authors:** Krzysztof B. Beć, Christian W. Huck

**Affiliations:** Center for Chemistry and Biomedicine, Institute of Analytical Chemistry and Radiochemistry, Leopold-Franzens University, Innsbruck, Austria

**Keywords:** near-infrared, NIRS, spectra simulation, theoretical spectroscopy, anharmonic methods

## Abstract

Near-infrared (12,500–4,000 cm^−1^; 800–2,500 nm) spectroscopy is the hallmark for one of the most rapidly advancing analytical techniques over the last few decades. Although it is mainly recognized as an analytical tool, near-infrared spectroscopy has also contributed significantly to physical chemistry, e.g., by delivering invaluable data on the anharmonic nature of molecular vibrations or peculiarities of intermolecular interactions. In all these contexts, a major barrier in the form of an intrinsic complexity of near-infrared spectra has been encountered. A large number of overlapping vibrational contributions influenced by anharmonic effects create complex patterns of spectral dependencies, in many cases hindering our comprehension of near-infrared spectra. Quantum mechanical calculations commonly serve as a major support to infrared and Raman studies; conversely, near-infrared spectroscopy has long been hindered in this regard due to practical limitations. Advances in anharmonic theories in hyphenation with ever-growing computer technology have enabled feasible theoretical near-infrared spectroscopy in recent times. Accordingly, a growing number of quantum mechanical investigations aimed at near-infrared region has been witnessed. The present review article summarizes these most recent accomplishments in the emerging field. Applications of generalized approaches, such as vibrational self-consistent field and vibrational second order perturbation theories as well as their derivatives, and dense grid-based studies of vibrational potential, are overviewed. Basic and applied studies are discussed, with special attention paid to the ones which aim at improving analytical spectroscopy. A remarkable potential arises from the growing applicability of anharmonic computations to solving the problems which arise in both basic and analytical near-infrared spectroscopy. This review highlights an increased value of quantum mechanical calculations to near-infrared spectroscopy in relation to other kinds of vibrational spectroscopy.

## Introduction

Near-infrared spectroscopy (commonly abbreviated as NIRS) has distinguished itself by a remarkable evolution from an undervalued section of vibrational spectroscopy to one of the presently most widespread modern analytical techniques with strong prospects for further expansion (Ciurczak and Drennen, 2002; Siesler et al., 2002; Cozzolino, 2014; Huck, 2014, 2016a; Ozaki et al., 2017). The second major application field taking advantage of the potential embodied in NIRS is hyperspectral imaging (Ozaki et al., 2017; Türker-Kaya and Huck, 2017; Dorrepaal and Gowen, 2018; He et al., 2018), which enjoys a rapidly growing interest in the biomedical applications (He et al., 2015; Türker-Kaya and Huck, 2017) and is a subject of focused development nowadays (He et al., 2015; Sun et al., 2017; Wong et al., 2019). As will be discussed further on, most of the distinctiveness of NIRS results from the specificity of the corresponding spectral region located between visible and infrared (near-infrared or NIR; 800–2,500 nm; 12,500–4,000 cm^−1^). From the point of view of the present review, the foremost aspect worth emphasizing is the molecular mechanisms standing behind the absorption of electromagnetic radiation in NIR, which involves excitations of non-fundamental vibrations, overtones and combination modes. The primary parameters of the resulting bands, wavenumbers and intensities are ruled by anharmonic effects with inter-mode anharmonicity playing the most substantial role. In this sense, NIRS distinctively sets itself apart from the other kinds of vibrational spectroscopy (mid-infrared, MIR; far-infrared, FIR; Mantsch and Naumann, 2010,^1^ and Raman) in which the major chemical information originates from fundamental vibrational transitions. Unlike these latter ones, which put the harmonic approximation into good use, theoretical NIRS unequivocally requires computationally intensive anharmonic approaches. Hence, NIR spectral simulations have remained rather rare in literature until the recent advances in theory and computer technology made such studies feasible for the molecules extending beyond a few atoms in complexity. It can be stated that this is an emerging field, as the entirety of literature reporting on theoretical NIR simulations of the molecules relevant for applied studies has emerged in the current decade.

The aim of the present review is to provide a comprehensive introduction to theoretical NIRS and to expound its distinctiveness and exceptional significance for the contemporary progress of applied spectroscopy (Graphical Abstract). In order to fully apprehend a remarkable synergy arising between the theoretical and experimental NIRS, far outweighing the analogous relation existing in the other kinds of vibrational spectroscopy, the present article includes brief information on the specificity of basic and analytical NIRS. Throughout the following sections, the underlying phenomena and key correspondences are thoroughly explained, together with a brief history sketch reaching the most recent accomplishments and future prospects, outlining a complete overview of the highly promising and boundary-crossing development currently taking place in the field of NIRS.

**Graphical Abstract:**
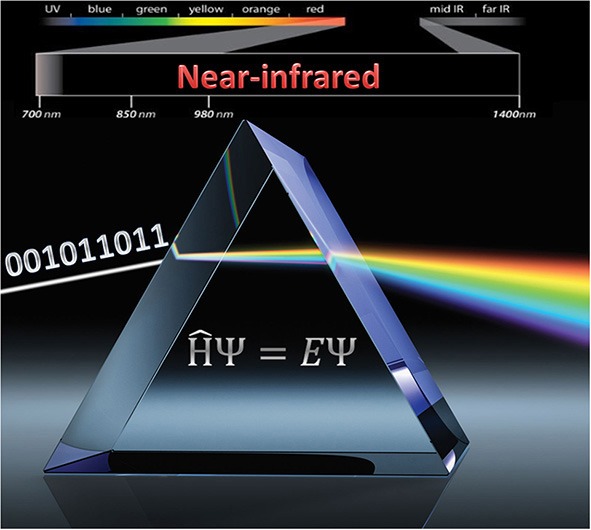
The properties of near-infrared spectroscopy create unique synergy with quantum mechanical spectra simulations.

## Near-Infrared Spectroscopy. The Tale of An Ugly Duckling

### Early Developments

**Figure 1:**
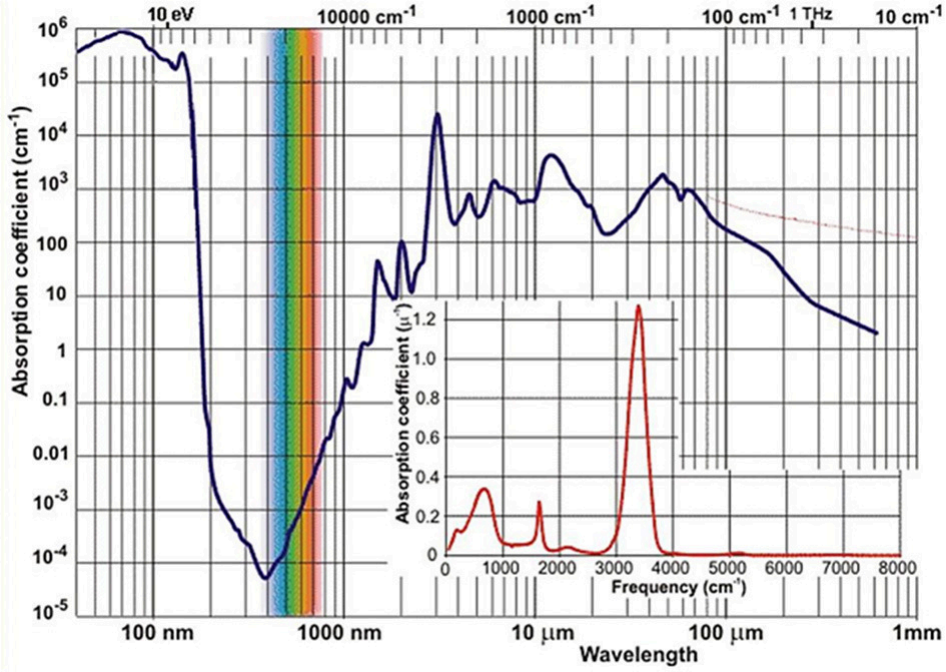
The spectrum of absorption coefficient of liquid water^2^.

Near-infrared light has been the first kind of non-visible electromagnetic radiation discovered as early as in year 1800 in the famous experiment of Herschel. Although this happened by observing that NIR light is absorbed by matter, it may be considered a peculiar plot twist of scientific history that the actual development of NIR spectroscopy has lagged behind the techniques resorting to other spectral regions, e.g., ultra-violet (UV), visible (VIS) or MIR. By the time these other kinds of optical spectroscopy have reached reasonable levels of maturity (ca. 1950–1960s), instrumentation capable of working in the NIR region was still being used only as an add-on unit to the major optical devices. The potential of NIR region remained not recognized at that time for several reasons. First, in basic research the lack of stimulus resulted primarily from the competition from MIR spectroscopy. The chemical information derived from the fundamental bands has always seemed more specific and readily available. The absorption occurring in the NIR region of the vast majority of both organic and inorganic matter is relatively weak. An example of a common and relatively strongly absorbing medium is liquid water, whose NIR absorption coefficient values remain at least two orders of magnitude lower than in the MIR region (Figure 1). At that time, before the era of Fourier-transform (FT) optical spectrometers, the relatively primitive dispersive devices have, by design, been less potent in capturing weaker signals. Both the registered peak positions and absorption intensity values have been rather unreliable because of the imperative instrument calibration and also due to analog electronics (Davies, 2011). The spectra collection was a time-consuming and manpower-intensive process. Additionally, weak and broad structures resulting from multiple overlaying bands are typically found in this spectral region. Hence, NIR absorption was troublesome in measurement and analysis and its potential has long remained undervalued (Davies, 2011).

In this context, the fact that analytical chemistry first reached to the unique favors of NIR spectroscopy may be unexpected. Briefly during the 1950s, the NIR region attracted limited attention in measurements of moisture in hazardous materials, where MIR spectroscopy found limited applicability due to strong absorption of water (Figure 1). However, other emerging analytical techniques (e.g., chromatography) proved to be even more superior for such a task and soon took over. Much has changed when an engineer K. Norris working for the United States Department of Agriculture re-invoked NIR spectroscopy for the analysis of moisture in grains and oilseeds (Davies, 2011). This required the designing of an instrument featuring a very high light efficiency. Over the next years, Norris and collaborators further developed early analytical NIRS, often facing scientific and engineering challenges, as well as straight opposition from some part of the spectroscopic community. Although early instrumentation has initially been troublesome and barely reliable enough for analytical applications, the unique advantages of applied NIR spectroscopy have been recognized and this activity marked the beginning of the evolution path leading to the present state-of-the-art NIRS (Davies, 2011).

### Analytical NIRS Today

Nowadays NIRS is being widely used in modern analytical applications due to a uniquely synergistic combination of qualities. Its universality, wide applicability, uncomplicated instrumentation, low time-to-result and low cost factors are prominent advantages from the point of view of qualitative and quantitative analysis (Ciurczak and Drennen, 2002; Siesler et al., 2002; Cozzolino, 2014; Huck, 2014, 2016a; Ozaki et al., 2017). It enables the non-invasive, non-destructible analysis of a variety of samples while maintaining a good balance between its cost, time, and analytical performance. The mentioned typical low absorptivity in NIR (Figure 1) results in the ability of examining a bulk sample in high volume with no limitation to the sample surface, as often encountered in optical spectroscopy. NIRS has found its way to quality control laboratories dealing with, e.g., food (Smyth and Cozzolino, 2013; Henn et al., 2016; Ringsted et al., 2017; Chapman et al., 2018) and natural products (Pezzei et al., 2017a), agriculture-related items (Pezzei et al., 2017b), pharmaceuticals (Kirchler et al., 2017a; Yan and Siesler, 2018a), phytopharmaceuticals (Stecher et al., 2003) and phytoanalysis in general (Huck, 2017a), polymers (Huck, 2016b; Unger et al., 2016; Yan and Siesler, 2018b) fuel (Lutz et al., 2014a), cosmetics^2^ (Blanco et al., 2007), biomedical applications (Jue and Masuda, 2013), general industry (Huck, 2017b) and environmental studies (Altinpinarn et al., 2013; Roberts and Cozzolino, 2016, 2017), among others (Ciurczak and Drennen, 2002; Siesler et al., 2002; Iwamoto, 2009; Cozzolino, 2014; Huck, 2014, 2016a; Ozaki et al., 2017; Power et al., 2018; Yan and Siesler, 2018c). In response to strong demand from the industry the instrumentation has been undergoing continuous development. One of the most recent milestones in this evolution path was the introduction of portable spectrometers (Herberholz et al., 2010; O'Brien et al., 2012; Alcalà et al., 2013), which marked the beginning of truly on-site capability of NIRS analysis (Wiedemair et al., 2018). Within the next decade, strong prospects exist for this technique to ultimately achieve wide-spread use even at consumer level due to the development of inexpensive mobile phone-attachable spectrometers and highly autonomous software for data analysis (Klakegg et al., 2016; Watanabe et al., 2016). The cost factor should not be underestimated, as it is a crucial factor for wide utilization; this is a subject of focused development (Saranwong et al., 2013). It may be reasonably envisioned that the next major breakthrough will be accomplished by the successful design and implementation of single-chip *in-silicon* spectrometers; the era of such devices in MIR spectroscopy is approaching (Wang et al., 2013; Ribessi et al., 2016; Sieger and Mizaikoff, 2016) and similar evolution in NIRS should be anticipated.

Analytical NIRS relies extensively on various methods of statistical analysis, which are commonly grouped under the well-established term of chemometrics (Beebe et al., 1998; Marini, 2013). These methods may be roughly divided into three classes. (1) Exploratory Data Analysis (EDA) includes techniques of data mining (e.g., Cluster Analysis, PCA-Principal Component Analysis) which are used for gaining deeper insights into high-volume complex data such as a large set of NIR spectra. (2) Regression analysis groups the methods used for the prediction/quantification of chemical content (predictive models); it finds extensive use in detection and quantification of selected chemical components. The most utilized techniques include Multiple Linear Regression (MLR), Principal Component Regression (PCR) and Partial Least Squares Regression (PLSR). (3) Classification techniques are used for the separation and sorting as well as grouping of samples with regard to a selected property. Classification approaches include supervised (e.g., SIMCA, Soft Independent Modeling of Class Analogy; LDA, Linear Discriminant Analysis; PLS-DA, Partial Least Squares Discriminant Analysis or SVMC, Support Vector Machine Classification) and unsupervised approaches (e.g., K-mean and K-median methods, Hierarchical Cluster Analysis or PCA, this time in its classification role). Classification methods allow e.g., group samples in accordance with their source of origin, level of authenticity, or even the region or conditions of cultivation in the case of agricultural products. Within the set content rule, the classification methods may be used, for example, for the separation of contaminated samples from the pure ones.

At present, analytical NIRS is an active research field with a considerably wide scientific and professional community involved, as evidenced by the narrowly scoped and highly attended international conferences (to list only the few: meetings of International Council for Near-Infrared Spectroscopy [ICNIRS]; International Diffuse Reflectance Conference [IDRC]; Asian NIR Symposia [ANS]), and scientific journals (e.g., *Journal of Near Infrared Spectroscopy*; *NIR News*). The development of the data analytical/statistical methods (i.e., chemometrics Burns and Ciurczak, 2007), which are essential in analytical NIRS, has grown to become another largely independent field of research (e.g., *Journal of Chemometrics*; *Chemometrics and Intelligent Laboratory Systems*; *Journal of Multivariate Analysis*). On the other hand, a rapid expansion of applied NIRS has resulted in its detachment from basic spectroscopy, physical chemistry, and molecular science. This fact, in turn, creates hindrances in the further advancement of this field, as will be explained in detail below.

The stimulated development of applied NIRS created focus on relatively narrow, short-reaching goals suiting specific analytical applications. This pragmatic demeanor has led to a detachment of analytical NIRS from its physicochemical background. Basic NIR investigations of the chemical structure, molecular vibrations, and intra- and inter-molecular interactions remain limited in relation to the exceedingly active field of applied studies (Huck, 2014). Due to an intrinsic complexity of NIR spectra, reflecting the anharmonic nature of molecular vibrations (Huck, 2014; Ozaki et al., 2017) the observed spectral outline typically emerges from multiple overlapping contributions. The resulting broad and non-homogeneous bands create significant difficulties in the comprehensive association of the observed spectral variability and physicochemical background. For this reason, in NIR analytical routines, spectral data is often used without any attempt to interpret the chemical information embodied within. Chemometrics allows the correlation of subtle spectral variability with the sample composition and thus yields effective chemical content detection and quantification. However, it lacks in providing a physicochemical interpretation for the information it delivers. In this context, the analytical NIRS is often effectively limited to a “black-box tool.”

### NIRS in Physical Chemistry

The value of NIRS to basic research stems from specific physicochemical features differing from those of the fundamental region (IR, Raman) (Czarnecki et al., 2015). NIR spectra remain the most natural and rich source of information on the anharmonicity of molecular vibrations. The absorptivity of NIR transitions gradually decreases toward the higher tones and higher order combinations. The co-existence of various bands (e.g., first, second, and third overtones Gonjo et al., 2011; Futami et al., 2012; Chen et al., 2014) within the same spectra is a key advantage here. Low band intensities enable systematic studies of the molecules in solution with a widely spread concentration range. This provides the possibility of a comprehensive investigation of intermolecular interactions, association mechanisms, solvent effects, molecular self-organization, and the structure of liquid phase (Czarnecki et al., 2015; Wrzeszcz et al., 2016a,b). In the NIR region, the bands originating from X-H (e.g., C-H, O-H, N-H) vibrations are strongly articulated. These chemical groups are most commonly responsible for the formation of hydrogen bonding. This enhances the potential of exploring the nature of hydrogen bonding and molecular interactions (Czarnecki et al., 2015). In the NIR region, particular types of bands are being enhanced in their intensity. The bands due to non-associated species usually are much more intense than those of aggregated molecules (Czarnecki et al., 2015). The NIR band heights often carry valuable information on themselves. For example, the prominence of the first overtone band of C=O stretching mode (5,260–5,130 cm^−1^) varies strongly among different molecular systems (Czarnecki et al., 2015). On the other hand, the second overtone of C=C stretching mode is not commonly observed in NIR spectra. One can conclude on this mode by investigating the spectral shift of its first overtone band or the combination band with the modes of a C-H group connected directly to the C=C moiety. Another relevant example may be provided in the form of the first overtone of C≡N stretching mode, or the second overtone of C=N mode. Contrary to the respective fundamentals, these transitions have very low absorptivity and have not yet been identified in the NIR region.

The specificity of such vibrational effects in NIR, different from those observed in MIR spectra, create a large amount of independent spectral information of high value for physical chemistry. However, NIR spectral analysis remains prone to ambiguities due to overlapping, anharmonicity, and the omni-presence of coexisting effects, which translate to convoluted spectral changes. Similar reasons have also been forming a hindrance in analytical NIRS. The intrinsic complexity of the spectra has forced the extensive usage of spectral pretreatment methods and advanced data analysis, for example, two-dimensional correlation spectroscopy (2D-COS) (Noda, 1989; Noda et al., 1995; Noda and Ozaki, 2004). The 2D-COS technique allows the expanding of the spectral information onto two-dimensions, basically elucidating the correlations in the spectral variations and visualizing straightforwardly the complex dependencies which would be otherwise difficult to trace in linear NIR spectra. It finds an extreme value in the analysis of spectral variations, as not only the changes are illuminated, but their direction and sequence can also be elucidated. 2D-COS also gains a better resolution through the simplification of the spectral data, the possibility of a more distinct assignment of bands, and exploration of the sequential order of changes occurring in the sample. Therefore, the different inter- and intramolecular behavior of samples can be investigated through the peaks visible in the synchronous (*syn*) and asynchronous (*asyn*) spectra, which are obtained by spreading spectral information as a function of two independent wavenumber axes over the second dimension (Noda and Ozaki, 2004).

A brief note on NIRS in biophysical chemistry should be made here as well (Huck, 2016a). In addition to the advantages outlined above, low absorptivity of NIR radiation also promotes its value when applied for the purpose of hyper spectral imaging (Huck, 2016a; Ozaki et al., 2017; Türker-Kaya and Huck, 2017; He et al., 2018). The resulting relatively deep penetration of NIR light plays a key role here, allowing an effective in-depth probing of the sample, a fact which finds an exceptional applicability in biophysical and biomedical studies (Huck, 2016a; Sun et al., 2017; Türker-Kaya and Huck, 2017). This currently remains a strongly developing area (He et al., 2015; Dorrepaal and Gowen, 2018; Wong et al., 2019), ultimately aiming at multi-modal imaging (He et al., 2018), which gives rise to an intriguing question of how in the nearest future this may be hyphenated with a similarly rapidly growing theoretical NIRS. This review will also include a short exploration of this topic.

## Theoretical Near-Infrared Spectroscopy–An Overview of the Emerging Field

### Fundamentals of Theoretical NIRS

As mentioned earlier, the simplistic harmonic approximation of molecular vibration brings substantial practical advantages from the point-of-view of computational complexity. The Newton equations of molecular oscillation lead to a matrix eigenvalue equation in which the harmonic vibrational frequencies are obtained through diagonalization of the matrix of mass-weighted second derivatives of the potential energy (mass-weighted Hessian). Consequently, the transition intensity is calculated from the derivative of dipole moment over the normal coordinates. Since Hessian is given as a straightforward output of the geometry optimization procedure, harmonic frequencies are calculated with a relatively minor additional cost. A commonplace overestimation of harmonic frequencies is being routinely addressed with a simple empirical scaling, thus yielding cost-effective theoretical MIR or Raman spectra (Wilson et al., 1955). However, NIR modes unequivocally require to step beyond harmonic approximation. While di-atomic anharmonicity remains relatively easy to account for, the problem arises in complexity for polyatomic molecules, mostly due to mode-mode couplings. A variety of anharmonic approaches has been proposed in the literature. However, in practical terms these methods have been prohibitively expensive and thus, until recently, have not been employed for the prediction of NIR spectra of reasonably complex molecules. The computational affordability is the primary factor from the point of view of applied spectroscopy. The anharmonic approaches may thus be categorized by their accuracy vs. cost ratio. In this sense, variational methods may be omitted due to extreme expense. This holds, in spite of their capability to yield an exact solution, limited only by the quality of potential energy evaluation. Variational methods are practically applicable to the simplest systems only (Polyansky et al., 2003). In contrast, applied spectroscopy relies on reasonably efficient anharmonic methods with the ability to treat complex molecules at a controlled penalty of a less accurate description of certain non-essential factors. For example, the Vibrational Self-Consistent Field (VSCF) method, which has fairly often been used for anharmonic simulation of MIR spectra (Gerber et al., 2005; Lutz et al., 2014b), assumes full factorizability of vibrational wavefunction into a set of normal mode wavefunctions. However, the VSCF scheme requires reasonably accurate probing of the vibrational potential; it is commonly based on a 16-point grid. The key economical feature here is the approximate treatment of inter-modal anharmonicity, effectively in which any given mode feels an averaged effect resulting from all other modes. This approximation has often been too severe; a number of refinements in the approach appeared, in which the cost-accuracy balance has frequently been skewed toward accuracy. For example, an improved variant, perturbation-corrected VSCF (PT2-VSCF) uses the second-order perturbation theory to correct the VSCF level couplings, yielding a higher certainty (Jung and Gerber, 1996). Concurrently, a number of reported attempts have been aimed at increasing the method's affordability, e.g., by reducing the grid-density for potential evaluations (Lutz et al., 2014c) or by employing more efficient ways for the determination of the electronic structure underlying the layer of anharmonic vibrational analysis [e.g., the resolution of the identity (RI) approximation in connection with the Moller-Plesset second order perturbation, i.e., RI-MP2; (Lutz et al., 2015)]. Interestingly, the precision penalty of basic VSCF decreases as the molecule complexity increases. This occurs due to a more effective averaging with the increasing numbers of modes within the VSCF mean-field approximation and has been exploited, for example, in biomolecule studies (Pele and Benny Gerber, 2010).

Another class of anharmonic methods is based on the vibrational second-order perturbation theory (VPT2) (Nielsen, 1951; Clabo et al., 1988). In principle, these include the anharmonic correction of vibrational potential in the form of cubic and quartic force constants yielded through the numerical differentiation of the harmonic Hessian at molecular geometries only slightly displaced from the equilibrium; very few energy evaluations are needed in this case. VPT2 methods are known for being computationally cost-effective; this is particularly evident if only bi-modal correlations are included in the calculations. However, the applicability of these methods has typically been hindered by their tendency to produce meaningless results in case of tightly-coupled modes (close degeneracies, i.e., vibrational resonances). The appearance of such singularities does not follow any consecutive pattern. Instead, their presence depends on a particular molecule; consequently, customized solutions have typically been needed almost each time. This fact severely restricted VPT2 usage in applied spectroscopy. Attempts have been made to develop an automated treatment of close degeneracies (Barone, 2005). For example, deperturbed (DVPT2) and generalized (GVPT2) schemes have appeared recently. These methods identify and remove close degeneracies from the perturbative treatment (in DVPT2 scheme) and reintroduce the removed terms through the variational approach (only in GVPT2 approach) (Barone, 2005). Essential for applied spectroscopy, these methods are suitable for any general molecule, effectively creating a robust and generally applicable tool for anharmonic calculations of even fairly complex molecules. Investigations into the solution phase often find a useful addition of computationally affordable implicit solvation model. Several different approaches may be found in recent literature, e.g., Polarizable Continuum Model (PCM) and derivatives; conductor-like polarizable continuum model (CPCM) or integral equation formalism variant (IEF-PCM); these approximate description of solvation allow for efficient increase in the quality of simulations performed in solution phase (Beć et al., 2016a, 2017).

Other anharmonic methods may also be mentioned, e.g., vibrational configuration interaction (VCI) (Whitehead and Handy, 1975) and vibrational coupled-cluster (VCC) (Christiansen, 2004) methods. These computationally highly expensive approaches are rarely found in the literature being used for the simulation of MIR spectra of simple molecules, particularly in cases where certain vibrational intricacies cannot be omitted (Oschetzki et al., 2013). Ongoing development in high-power computing may result in an increased application of these methods in the future. A final remark may be aimed at anharmonic calculations of macromolecules and biomolecules. As is known, anharmonic effects are sometimes substantial in those cases; certain biomolecules, e.g., proteins or nucleic acids, exhibit strong anharmonicity expressed in low-barrier bond torsions, low-energy vibrations in the THz region, ring modes in large ring systems, or hydrogen-bonded complexes (Hamm and Hochstrasser, 2001; Walther et al., 2002). In these applications, even a basic account for anharmonicity may yield significant gains. Due to the complexity of such molecules, the computational cost factor remains the center of attention. A number of efficiency-oriented methodological studies aimed at large molecular systems has emerged; this challenging research area meets strong stimulus from various fields and remains very active (Levy et al., 1984; Pele and Benny Gerber, 2010; Schlick, 2010; Krasnoshchekov and Stepanov, 2015).

Recent times have seen a progressing amount of quantum mechanical (QM) simulations of NIR spectra. Such studies, so far, have mostly utilized either VSCF or VPT2 routes, as those possess the required balance between accuracy and computing cost, essential in applied spectroscopy. Various examples of such studies will be overviewed beneath.

### Basic Molecules

**Figure 2:**
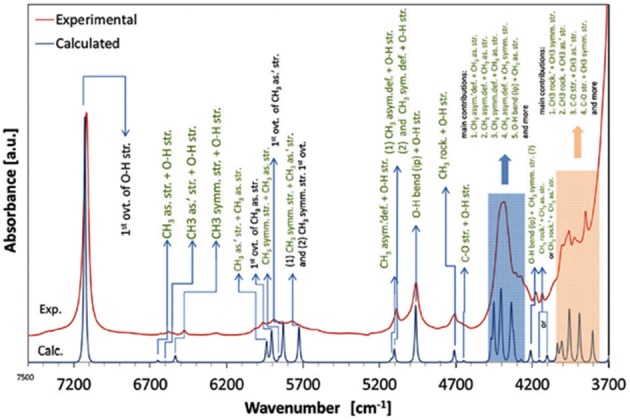
NIR spectra of diluted methanol; experimental (5 10^−3^ M CCl_4_) and simulated by the use of anharmonic calculations (GVPT2 scheme on DFT-B2PLYP/SNST level of electronic theory and CPCM solvation model of CCl_4_) (Beć et al., 2016a). Reproduced by permission of the PCCP Owner Societies.

**Figure 3:**
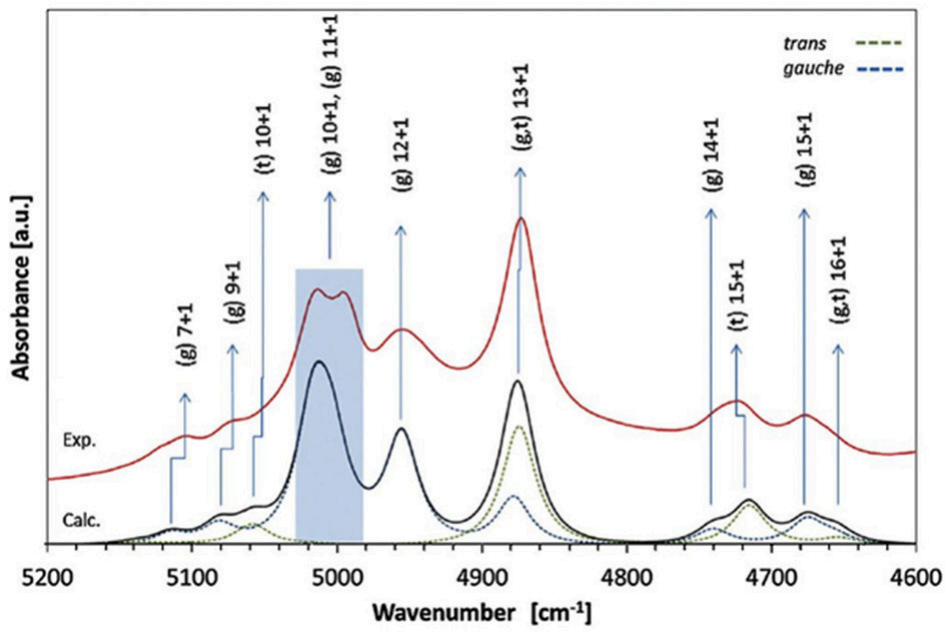
Band assignments in the experimental and calculated NIR spectra of low concentration (5 10^−3^ M CCl_4_) ethanol. The calculated NIR spectrum is based on the CPCM-B2PLYP-D/SNST level of theory. Details of the 5,200–4,600 cm^−1^ region (Beć et al., 2016a). Reproduced by permission of the PCCP Owner Societies.

A number of QM studies on NIR spectra of basic molecules have been reported over the last few years (Beć et al., 2016a, 2018a; Grabska et al., 2017a,b). Vibrational studies of these systems are relatively important for our understanding of the general spectra-structure correlations (Czarnecki et al., 2000; Wojtków and Czarnecki, 2006; Michniewicz et al., 2007, 2008; Haufa and Czarnecki, 2010) and the role of conformational isomerism (Czarnecki et al., 2006; Wojtków and Czarnecki, 2006), structure and dynamics of hydrogen-bonding, self-association mechanisms and intermolecular interactions (Czarnecki and Ozaki, 1999; Czarnecki et al., 2000, 2010; Czarnecki, 2003; Czarnecki and Wojtków, 2004; Michniewicz et al., 2007; Haufa and Czarnecki, 2010), in particular the interactions with solvent molecules (e.g., water, nonpolar solvents) (Gonjo et al., 2011; Tomza and Czarnecki, 2015), or chiral discrimination (Czarnecki, 2003); the temperature influence on the above effects was often also examined (Czarnecki and Ozaki, 1999; Iwahashi et al., 2000; Šašić et al., 2002; Wojtków and Czarnecki, 2005, 2006, 2007; Grabska et al., 2017a). In the above contexts, alcohol molecules have remained among those investigated most frequently in physicochemical NIRS; often, the lack of QM spectra simulations hindered full comprehension therein. Beć et al. recently investigated basic alcohols; methanol, ethanol and 1-propanol by experimental and theoretical NIRS, demonstrating the potential of such approach in gaining a deep understanding of these spectra (Beć et al., 2016a). A good agreement between the experimental (solution; 3 10^−5^ M in CCl_4_) and calculated spectra has been achieved, including the reproduction of finer bands (Figures 2–3) and the manifestation of conformational isomerism. This demonstrated well the potential of QM spectra simulation in explaining the spectra-forming factors in NIR (Figure 2) far surpass that achievable by classical spectroscopic methods even for an elementary molecule such as methanol (Weyer and Lo, 2002). Ethanol and 1-propanol feature more vibrational bands, and additionally, have conformational isomers which results in distinct spectral signatures (Figure 3). Beć et al. have shown that with QM simulations it is possible to unambiguously follow the contributions from conformational isomers in the NIR region (Beć et al., 2016a). They also reproduced the bandshape details of the 2νOH band, a major peak in NIR spectra of alcohols. A homogenous structure of methanol is clearly set apart from ethanol and 1-propanol, accounting for their conformational flexibility. The simulation reflected well an increasing complexity of 2νOH bandshape due to the separation of the contributions stemming from different conformers (Beć et al., 2016a).

In the study of simple alcohol molecules, the deperturbed/generalized vibrational second-order perturbation theory (DVPT2/GVPT2) has been used at several levels of electronic theory (Beć et al., 2016a). A number of basis sets have been considered, as well as a selection of three different solvation models within self-consistent reaction field (SCRF) formalism. This allowed for an effective method validation accounting to their feasibility in accurate reflection of NIR modes; similar comparisons assembled for fundamental (IR) modes are frequent in literature yet remain very rare for non-fundamentals. Beć et al. concluded that the density functional theory (DFT) should be prioritized in applied spectroscopic studies. DFT offers a good cost-precision balance, this being a primary factor since anharmonic vibrational analysis is notably expensive in itself (Table 1). Post Hartree-Fock approaches, e.g., the Møller-Plesset second order perturbation (MP2) method may find limited use due to unfavorable accuracy/computing time factor. DFT with a carefully selected density functional, matching the complexity of the calculated molecule, may be considered the best choice here. For the treatment of molecules in solution, B3LYP and B2PLYP functionals are recommended, even more with the addition of empirical correction for dispersion improving the description of non-covalent and long-range interactions at a moderate cost. The conclusion was that double-hybrid functionals, e.g., B2PLYP, offer better consistency of NIR frequencies albeit when short-time of the simulation is prioritized, single-hybrid B3LYP is sufficiently good. A more detailed evaluation of the computational demands of different electronic approaches used inVPT2 calculation has been reported by the same authors (Beć et al., 2016b). Calculations employing the B2PLYP functional were roughly twice as expensive as those with B3LYP; a triple-ζ SNST basis set versus a small double-ζ N07D basis increases the computing time by a factor of two, at least in the case of methanol molecule. Contrarily, an addition of the empirical dispersion correction and implicit solvation model (CPCM, IEF-PCM or SMD) introduce only a meager overhead (Table 1). Therefore, including these two supplementary calculation steps may be recommended; the former is applicable in general, while the latter is applicable to molecules in the solution phase (Beć et al., 2016a).

**Table 1:** An exemplary comparison of total computational time for methanol molecule (including geometry optimization, harmonic calculations and VPT2 treatment).

**Method**	**Solvent model**	**CPU time**^a^**[s]**	**Wall time**^a^**[s]**	**Wall time relative ratio**
B3LYP/6-31G(d,p)	–	117	353	1
B3LYP/6-31G(d,p)	CPCM	135	375	1.1
B3LYP/6-31G(d,p)	IEF-PCM	147	409	1.2
B3LYP/6-31G(d,p)	SMD	151	422	1.2
B3LYP/N07D	–	157	466	1.3
B3LYP/SNST	–	246	1,345	3.8
B3LYP-D3/SNST	–	246	1,351	3.8
MP2/SNST	–	305	878	2.5
B2PLYP/N07D	–	370	1,060	3.0
B2PLYP/SNSD	–	660	1,860	5.3
B2PLYP/SNST	–	886	2,534	7.2
B2PLYP-FC/SNST	–	887	2,448	6.9
B2PLYP-D/SNST	–	891	2,520	7.1
B2PLYP/SNST	CPCM	928	2,515	7.1
B2PLYP-D/SNST	CPCM	938	2,644	7.5
B2PLYP/SNST	SMD	957	2,580	7.3
B2PLYP/SNST	IEF-PCM	971	2,623	7.4
MP2/aVTZ	–	2,512	6,553	18.6
MP2/aVQZ	–	46,083	119,288	337.9

a *The CPU time and wall time depend on the hardware platform. The presented values are for 24 core Intel Haswell architecture computing node. Reprinted with permission from Beć et al. (2016b)*.

**Figure 4:**
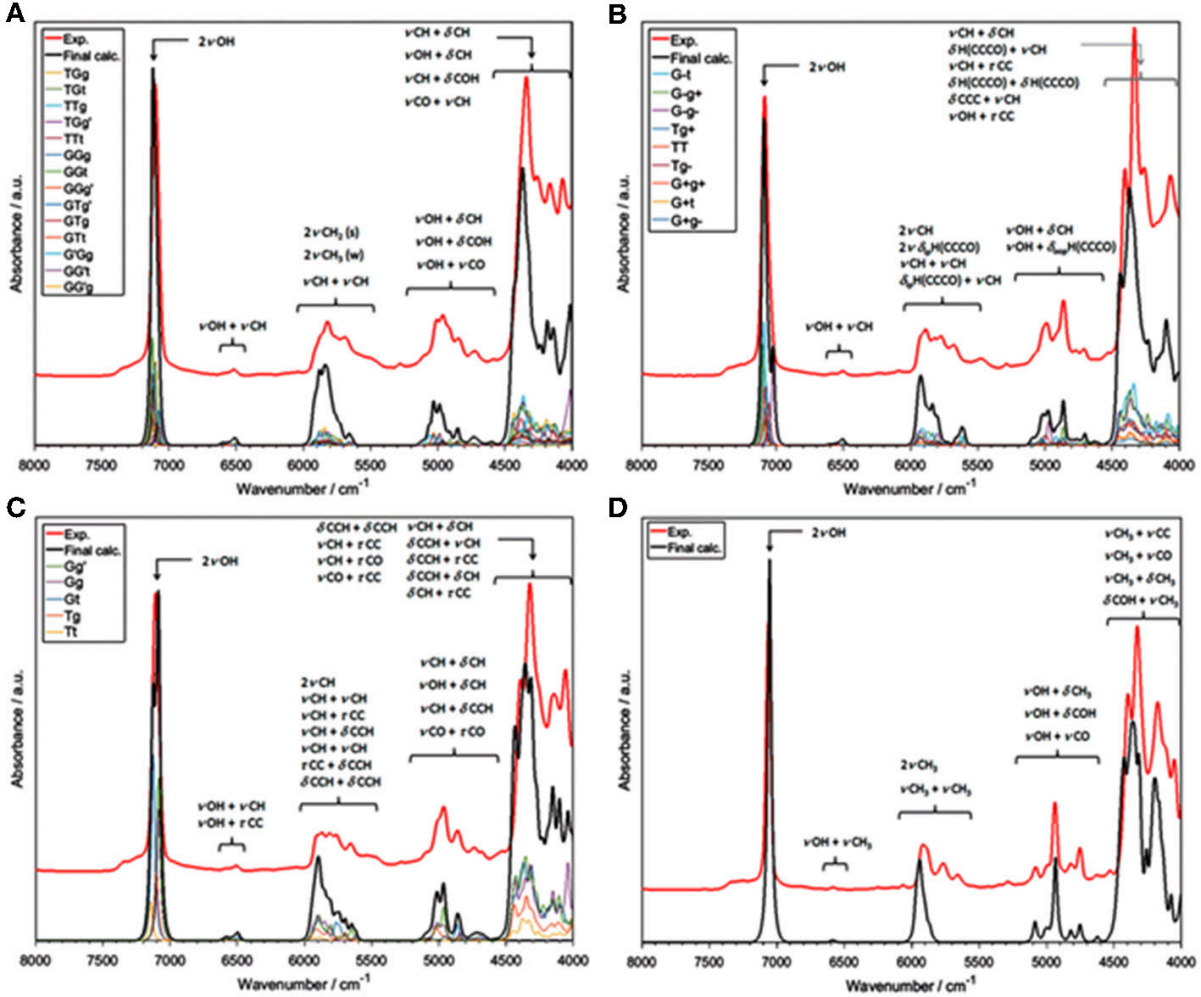
Experimental and simulated (harmonic: B2PLYP/def2-TZVP; VPT2: B3LYP/SNST; CPCM) NIR spectra of butyl alcohols; **(A)** 1-butanol; **(B)** 2-butanol; **(C)** *iso*-butanol; **(D)** *tert*-butyl alcohol. The contributions of the spectral lineshapes corresponding to conformational isomers presented as well (colored lines). Reprinted with permission from Grabska et al. (2017a). Copyright 2017 American Chemical Society.

The influence of temperature on the structure and association mechanisms of alcohols and similar molecules has always been a primary scientific problem studied in physicochemical NIRS. It has been well-known that in diluted alcohols the 2νOH band undergoes a temperature-induced spectral shift and intensity variation. Additionally, bandshape changes have been closely monitored by second derivation and two-dimensional correlation analyses (2D-COS); the latter technique has proven to be particularly powerful in these studies. In this context, examinations of butyl alcohols were helpful due to the differences among these isomeric molecules (Maeda et al., 1999; Czarnecki et al., 2015). A change of the 2νOH bandshape and a peakshift is observed in the case of 1-butanol, 2-butanol, and *iso*-butanol; however, *tert*-butanol spectrum features only a peak shift. Accordingly, 1-butanol, 2-butanol and *iso*-butanol feature conformational flexibility; on the contrary, *tert*-butyl alcohol is inflexible. These observations suggested that conformational isomerism should be responsible for the respective temperature-dependent NIR spectra inconsistency; yet, no decisive explanations could be provided at that time (Maeda et al., 1999). This problem has recently been reinvestigated by Grabska et al. this time with the solid support of QM simulations (Grabska et al., 2017a). Their attempt to theoretically reproduce the temperature induced spectral variations in the NIR region of butanols was successful. To improve the accuracy/cost balance in the anharmonic treatment of these larger molecules, they applied a hybrid approach in vibrational analysis; the harmonic and anharmonic steps were performed on different levels of electronic theory (B2PLYP/def2-TZVP and B3LYP/SNST; respectively), maximizing the efficiency of the calculation. The obtained simulated NIR spectra have resembled the experimental ones remarkably well (Figure 4), including the reproduction of subtle effects, e.g., minor bands between 5,200 and 4,500 cm^−1^ for all four butyl alcohols (Figure 4). Detailed and reliable band assignment and full comprehension of the NIR spectra of diluted butanols was achieved (Grabska et al., 2017a). In the next step, Grabska et al. investigated the temperature dependence of the conformational population of butyl alcohols (Grabska et al., 2017a). By calculating the Boltzmann coefficients corresponding to all conformational isomers of 1-butanol, 2-butanol and *iso*-butanol and subsequent use of these values in spectra simulation, including theoretical 2D-COS plots, they have succeeded in reproducing the temperature-dependent spectral shift and bandshape changes observed experimentally. This comparison has confirmed that the relative changes in the conformational populations contribute, at least partially, to the observed spectral variability. Hence, the background for the experimental observations was decisively explained and the old and never fully explained scientific problem was solved. Grabska et al. has adequately demonstrated the usefulness of QM simulations in bringing definite answers to the problems which have often been difficult to become unequivocally resolved by means of classical NIR spectroscopy (Grabska et al., 2017a).

**Figure 5:**
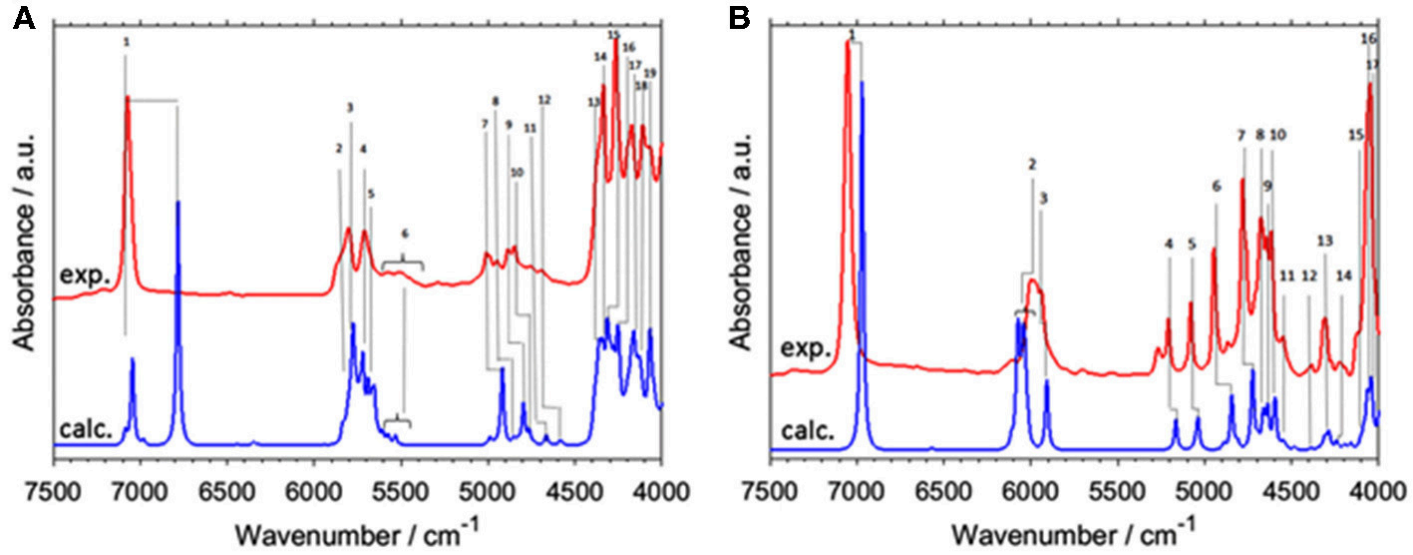
Experimental (0.2 M; CCl_4_) and simulated (VPT2//B3LYP/SNST+CPCM) NIR spectra of **(A)** cyclohexanol and **(B)** phenol. Reprinted with permission from Elsevier (Beć et al., 2018a).

A number of open questions related to NIR spectroscopy and physical chemistry of alcohols still remain. In example, Beć, Grabska and Czarnecki examined 1-hexanol, cyclohexanol, and phenol (Beć et al., 2018a). They focused on the spectra-structure correlations in NIR due to the vibrations of an OH group attached to a molecular skeleton of three different kinds; linear and cyclic aliphatic, and aromatic ring. It is well known that the bands due to X-H modes and particularly OH vibration, are enhanced in NIR spectra. Hence, OH vibrations belong to the primary spectra-forming factors; in this context, investigating how a molecular structure affects the NIR spectrum brings long-awaited answers (Beć et al., 2018a). Those three molecules manifest remarkable dissimilarity in NIR spectra; 1-hexanol remains similar to shorter chain linear alcohols. However, cyclohexanol, and even more phenol, had substantial differences (Figure 5). Detailed band assignments and the elucidation of distinct trends in NIR spectra could be carried out with the aid of QM simulations. The peculiarities of combination bands of νOH mode could be noted. This mode couples strongly to a number of other modes and gives a distinct spectral signature between 5,500 and 4,000 cm^−1^ (Figure 5). The specificity of phenol ring modes was also concluded. Good separation of the fundamental bands in the MIR region translates into NIR features with well-resolved sharp peaks appearing throughout lower wavenumbers (5,500–4,000 cm^−1^). This should be considered an uncommon observation in NIR spectra and also in elementary molecules e.g., methanol rather manifest broadened bands (Figure 2). The established signature allows to discriminate easily between different kinds of alcohols (aliphatic, aromatic) and to identify an OH group attached to an aromatic ring (Beć et al., 2018a). It should be noted that the accuracy of VPT2 simulation is lower for the 2νOH peak in general, but in the case of cyclohexanol it was by far inadequate (Figure 5A). The reasons for this are well-understood and will be discussed in detail in the present review.

### Investigations of Intermolecular Interactions

**Figure 6:**
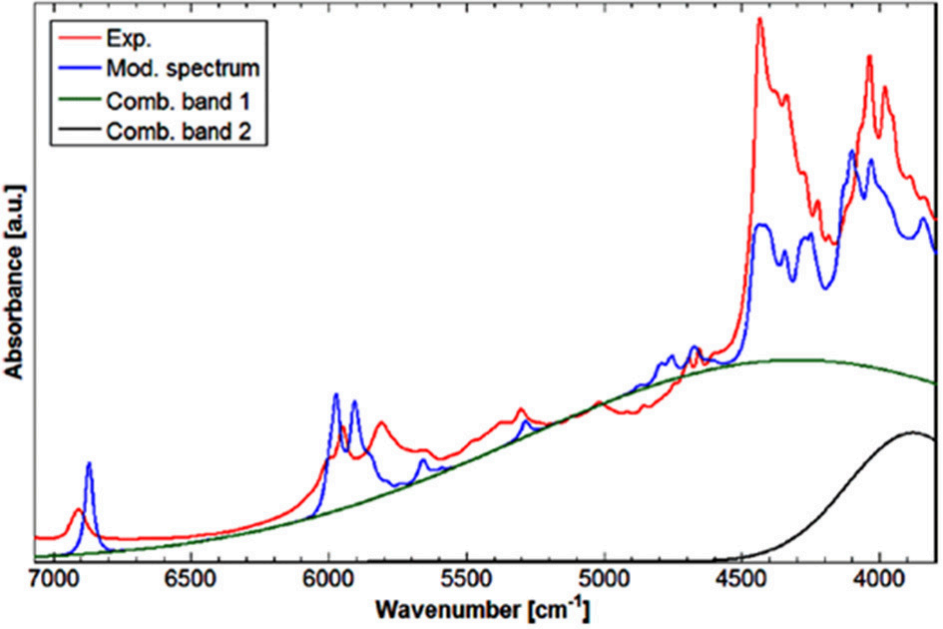
Experimental (solution; CCl_4_) and modeled spectrum of acetic acid. Band fitting results for the two combination bands involving OH stretching modes of acetic acid cyclic dimer. Reprinted with permission from Beć et al. (2016c). Copyright 2016 American Chemical Society.

NIRS manifests unique suitability for investigating intermolecular interactions, e.g., hydrogen-bonding. Not without reason, this aspect is strongly focused in physicochemical NIRS (Czarnecki and Ozaki, 1999; Czarnecki et al., 2000, 2010; Czarnecki, 2003; Czarnecki and Wojtków, 2004; Michniewicz et al., 2007; Gonjo et al., 2011; Tomza and Czarnecki, 2015). The potential of QM simulations has long been utilized in MIR and Raman studies. On the contrary, NIRS lacked such powerful support due to practical limitations, as explained earlier. The cyclic dimer of carboxylic acids, e.g., formic acid or acetic acid, has frequently been considered a prototypic system of the hydrogen bonded complex. Beć et al. have recently presented a combined experimental and computational NIRS study of acetic acid in a CCl_4_ solution (Beć et al., 2016c). The focus was on the spectroscopic properties of the cyclic dimer, which is strongly stabilized and persist as the major form throughout a widely variable concentration. The QM simulation has reproduced the majority of experimental NIR bands; however, a distinct exception was observed. The binary combination bands involving the stretching and bending of OH modes had a very strong calculated intensity, resulting in the appearance of two sharp and well-resolved peaks in the simulated spectrum. In contrast these are clearly absent in the experimental NIR lineshape. However, prominent elevation of the baseline is apparent in the NIR spectrum of soluted acetic acid, visible between 6,500 and 4,000 cm^−1^ and extending below. The following hypothesis was proposed in the article; the aforementioned combination bands undergo a spectral shift and broadening as a hydrogen-bonding effect; similar effects are well-known in MIR spectra. These two particular simulated combination bands were then fitted to the experimental spectrum to reflect the baseline contribution. Such treatment significantly improved the agreement with the experimental NIR spectrum (Figure 6). This investigation focused attention on the possibility of hydrogen bonding being manifested in the NIR spectrum through the baseline elevation (Beć et al., 2016c); such spectral feature is frequently observed in the NIR spectra of complex samples.

**Figure 7:**
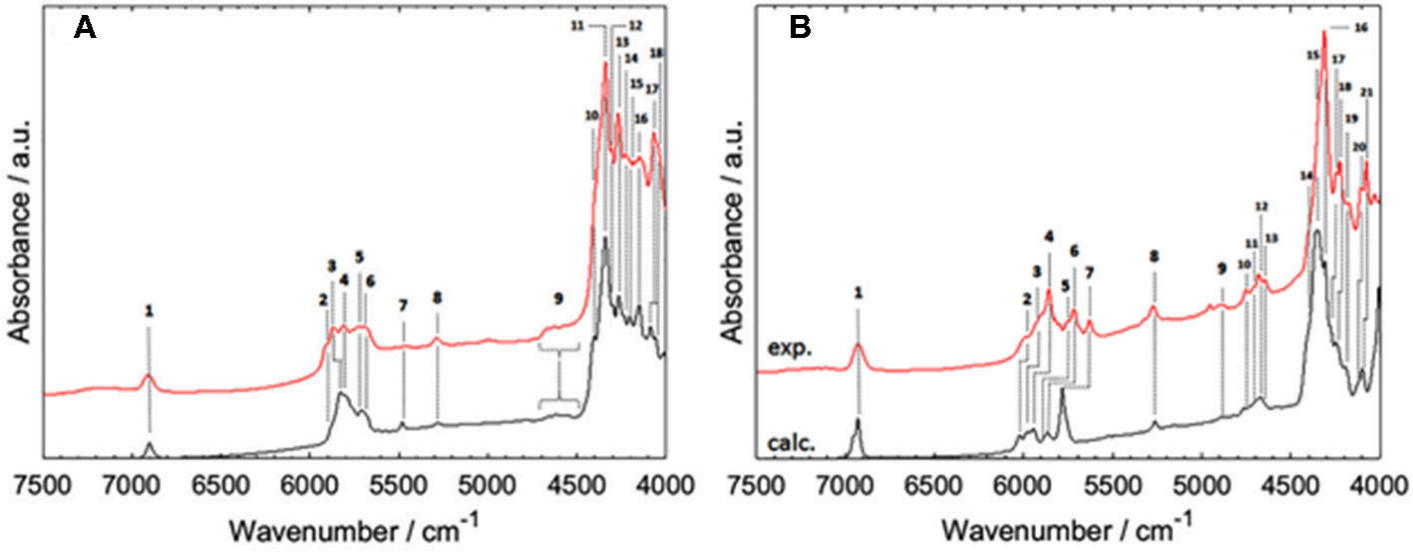
Band assignments proposed for NIR spectra of MCFAs in medium to high concentration (CCl_4_); **(A)** hexanoic acid, **(B)** sorbic acid. Reprinted with permission from Elsevier (Grabska et al., 2017d).

The basic study of acetic acid yielded a good understanding of NIR properties of simple carboxylic acids, which was helpful in the further exploration of fatty acids. These fundamental biomolecules can be grouped into short- (SCFAs), medium- (MCFAs) and long-chain fatty acids (LCFAs). Fatty acids manifest various properties interesting from the point of view of physical chemistry, e.g., association mechanisms and hydrogen bonding properties (Iwahashi et al., 1995a,b; Matsuzawa et al., 2013). These systems are also extremely important in applied NIR studies involving any kind of biological samples, e.g., in hyperspectral imaging and analytical applications (Ishigaki et al., 2016a,b; Puangchit et al., 2017). For these reasons, SCFAs and MCFAs were examined by Grabska et al. in their two following studies (Grabska et al., 2017c,d). The former one focused on five SCFAs; saturated: propionic and butyric acid; and unsaturated ones: acrylic, crotonic and vinylacetic acid (Grabska et al., 2017c). These carboxylic acids are reasonably more complex than acetic acid, and are thus appropriate test subjects for verifying if the conclusions on acetic acids can be generalized onto larger carboxylic acids. Additionally, their complexity and mutual differences in the structure are perfect for investigating the NIR manifestations of the aliphatic chain structure and the existence of C = C bonds (Grabska et al., 2017c). Grabska et al. have selected these objects to capture the principle structural features of FAs (Figure 7). Those included: the difference between saturated and unsaturated SCFAs, impact of the location of C = C bond (medium-chain: crotonic acid; terminal: acrylic and vinylacetic acid), exclusive existence of either of the three following structural features: methyl (crotonic acid), *sp*^3^ or *sp*^2^ (terminal; acrylic acid, vinylacetic acid), methylene group (Grabska et al., 2017c).

The DVPT2 anharmonic vibrational analysis performed at the B3LYP/SNST+CPCM level of electronic theory involved full conformational analysis for each of SCFAs in the spectra simulation procedure (Grabska et al., 2017c). The final agreement between the simulated and experimental spectra as measured in the solution phase (0.05 M; CCl_4_) was of high quality (Figure 9). The SCFAs case confirmed the previous observation; a baseline elevation phenomenon similar to that observed for acetic acid was clearly noticed (Beć et al., 2016c). Specific combination bands had higher intensities by orders of magnitude than those of the other NIR bands. Those combinations (*a* + *b* and *a* + *c*) result from the following modes: (a) out-of-phase (or opposite phase) stretching and (b) in-plane bending modes, each time combined with (c) in-phase stretching mode of hydrogen-bonded OH groups. Through a band-fitting procedure analogous to that performed for acetic acid, a much better reproduction of the experimental spectra was accomplished. The stimulus for attempting the band fitting resulted from an undoubtful absence of any intensive sharp peaks in the experimental spectra, which were suspected to form the baseline elevation through band broadening. Grabska et al. also observed consecutive trends throughout several NIR subregions of SCFAs. Specific disparities in overtone and combination intensities were observed; the elucidated contribution of the CH_3_ group was found to be fully consistent with experimental studies found in the literature. Unsaturated SCFAs revealed a specific impact on the NIR spectrum of the localization of C = C bond; e.g., terminal C = C imposing an appearance of sp^2^ CH_2_ group leads yields extremely specific, well-defined and intense NIR bands, observed at 6,172/6,131, 4,746/4,734, and 4,483/4,489 cm^−1^ for acrylic/vinylacetic acids, respectively. It was concluded that these bands are excellent structural markers because of high intensity and positioning in wavenumber regions where the bands of other structures remain absent (Grabska et al., 2017c).

A continuation of the above overviewed study was published soon after, in which the NIR features of two MCFAs, saturated hexanoic and unsaturated sorbic acid, were examined (Grabska et al., 2017d). Those molecules exhibit distinct differences in the NIR region observable from a very high dilution (minimal self-association) to a more concentrated solution (with the domination of the spectral bands from cyclic dimers; Figure 7). For both cases, accurate spectra simulation yielded a comprehensive explanation of the observed features. Evidence showed that the shape of NIR spectra is similar through a widely varying concentration; this holds even in neat liquid (hexanoic acid) and powder (sorbic acid) (Grabska et al., 2017d). These findings were consistent with previous ones, as reported for acetic acid and SCFAs (Beć et al., 2016c; Grabska et al., 2017c). The three discussed studies (Beć et al., 2016c; Grabska et al., 2017c,d) indicated that theoretical NIR biospectroscopy may become feasible as small and medium-sized biomolecules are within the potential for accurate NIR simulations. Further expansion onto more complex objects (e.g., LCFAs, lipids, proteins, nucleic acids) may be anticipated in the foreseeable future. So far, the reported accomplishments in theoretical NIRS of small biomolecules were successfully employed for the interpretation of NIR images of biosamples (Puangchit et al., 2017).

### Other Examples of NIR Simulations in Physical Chemistry

**Figure 8:**
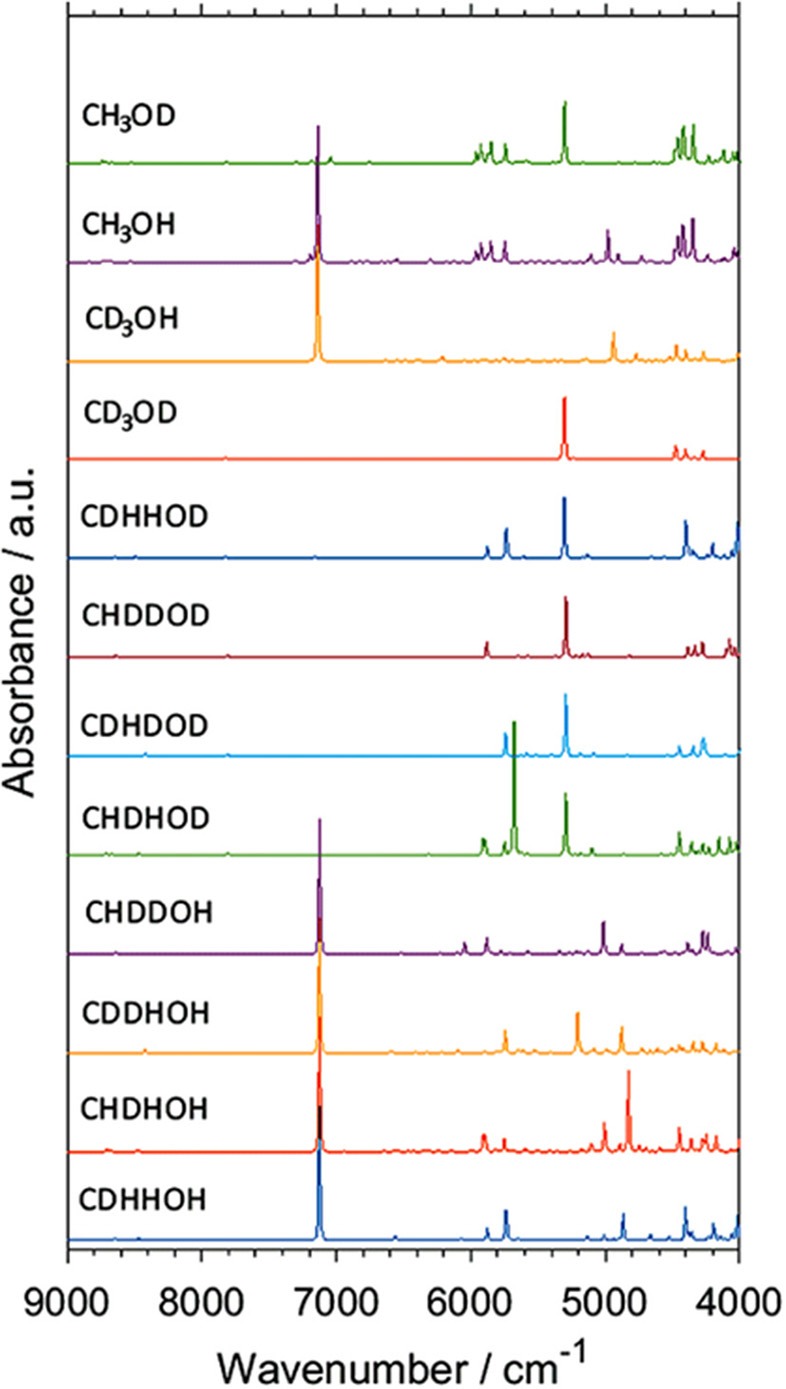
Simulated NIR spectra of CXXXOX (X = H, D) molecules. Reprinted with permission from Grabska et al. (2017b). Copyright 2017 American Chemical Society.

Isotopic substitution has definitely been a key phenomenon remaining in the center of attention of vibrational spectroscopy. It gives a prominent spectral signature; primarily significant spectral shifts (Jaffe, 1987; Davis et al., 1996; Workman and Weyer, 2007). For this reason, it has been one of the most potent tools of classical spectroscopy used for band assignments. One can relatively easily follow the spectral changes resulting from specifically arranged isotopic substitutions, e.g., through the deuteration of an OH or other functional groups in simple molecules. In this way, the corresponding spectral variability may be comprehended relatively easily even in the NIR region. However, an imperfect partial substitution (e.g., the existence of CX_3_ groups, where X = H,D are distributed randomly) resulting from a spontaneous isotope equilibration or faulty synthesis leads to significant difficulties in interpreting an NIR spectrum. Such random forms are impossible to be isolated from the sample, and thus, no reference spectrum of any of such forms can be recorded. In this case, spectra simulation is an extremely potent tool, as recently demonstrated by Grabska et al. In their comprehensive study of methanol and its deuterated derivatives, they examined all isotopomers of methanol molecule by simulating their NIR spectra (Figure 8; Grabska et al., 2017b). Through this, they successfully identified randomly substituted species found in two commercial samples of CH_3_OD, and they directly monitored different levels of contamination by random isotopomers of methanol molecule in NIR spectra. Such an achievement would be out of reach in classical spectroscopy. Additionally, the anharmonic QM simulation yielded comprehensive band assignments in the NIR spectra of the four major methanol isotopomers, CH_3_OH, CH_3_OD, CD_3_OH, and CD_3_OD. These compounds have routinely been employed in physicochemical NIRS. Grabska et al. also included vibrational transitions up to three quanta in their computational study. The resulting simulated spectra included first and second overtones as well as binary and ternary combinations (Grabska et al., 2017b). This move gave an opportunity to confirm an earlier assumed non-essential loss of spectral information in NIR spectra modeling when only first overtones and binary combinations are considered. The conclusion that a practical restriction of the simulation to two quanta transitions (first overtones, binary combinations) could earlier been only assumed, e.g., such simplification in the simulated spectra of butanols did not prevent an excellent agreement with the experimental bands (Figure 4). On this occasion, the study of methanol and deuterated derivatives confirmed that the loss of up to ca. 20% of spectral information may be anticipated. This estimation was based on the relationship between the summed integral intensity of the calculated bands. The lost (or omitted) calculated spectral information is distributed among multiple yet weak bands and remains effectively “diffused” over the wavenumber axis. Hence, second overtones and ternary combinations are not essential for NIR spectra comprehension. This approximation holds unless ones would want to examine the upper NIR region, over 7,000 cm^−1^. These wavenumbers have typically been rather rarely focused on in both physicochemical and analytical NIRS. However, upper NIR is the working region of some new miniaturized NIR instruments (Kirchler et al., 2017b). Thus, an additional ability to model second overtones and ternary combinations should be of increasing importance in the upcoming studies.

**Figure 9:**
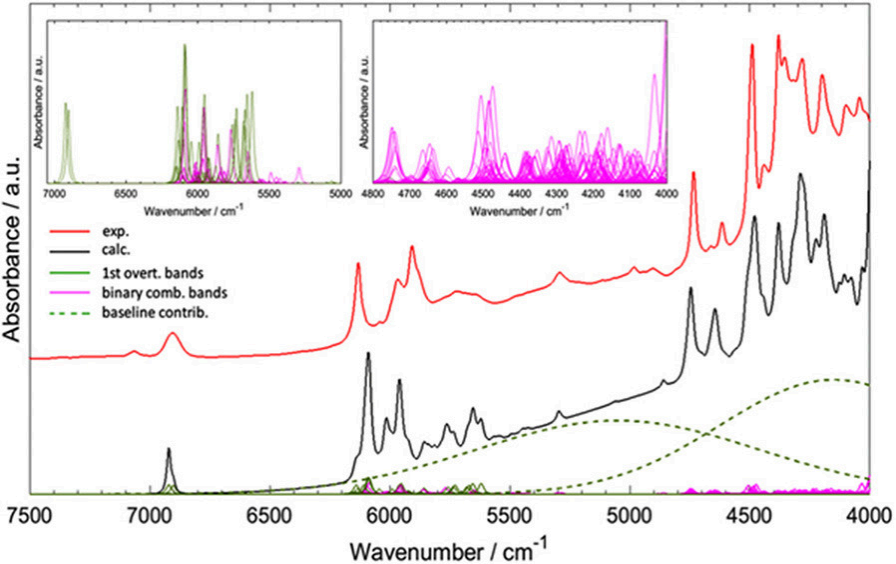
Convolution of NIR bands on the example of spectra simulation for vinylacetic acid. All bands are presented in common intensity, note an extensive band overlay. Reprinted with permission from Grabska et al. (2017c). Copyright 2017 American Chemical Society.

Worth emphasizing is the fact that QM calculations deliver a clear image of the intrinsically complex nature of NIR spectra. Although it has long been known, nowadays simulated spectra reproduce it accurately and visualize it straightforwardly. A good example of the NIR band overlay was provided by Grabska et al. in the case of vinylacetic acid (Figure 9; Grabska et al., 2017c). A common intensity scale of all bands depicted in Figure 9 adequately demonstrates the extensity of band overlapping. Despite the complexity of that and similar NIR spectra, QM simulation reproduced it accurately including very complex region of 5,000–4,000 cm^−1^, where the maximum overlay occurs due to appearance of the majority of binary combinations therein (Grabska et al., 2017c).

### Highly Accurate Modeling of Single-Mode Anharmonicity

Generally applicable anharmonic computational schemes, e.g., VPT2 or VSCF, through simplifications are affordable enough to be used for the calculation of a large number of transitions, e.g., first overtones and binary combinations of all modes (Beć et al., 2017). The general methods are highly useful in the simulation of an entire NIR spectra as overlapping of the manifold bands in this region would make it extremely difficult to rely on any arbitrary selection of particular modes. Exceptions can be found though, e.g., 2νOH band most often appears as a single peak at least in the absence of molecular association (Czarnecki et al., 2015; Beć et al., 2016a). At the same time, as a highly anharmonic mode it is frequently described inadequately by VSCF or VPT2 approaches. These methods acquire their efficiency by approximating the anharmonic potential on a possibly lowest number of energy evaluations. Thus, effectively they “capture” a limited amount of anharmonicity, which may be enough in most cases, but not necessarily, e.g., when the mode deviates from the harmonic oscillator to a certain degree (Beć et al., 2017). Several attempts to improve accuracy have been made e.g., by transformation of the vibrational coordinates; this remains an active field of research (Yagi et al., 2012; Thomsen et al., 2014). The application of VSCF or VPT2 calculations to highly anharmonic modes may yield unreliable results. Particularly troublesome cases may be addressed through a detailed study of the single-mode anharmonicity through multi-point energy evaluations, and solving the corresponding time-independent Schrödinger equation. With an adequately large number of evaluation points, the vibrational levels can be derived with very high accuracy (Gonjo et al., 2011; Yagi, 2016; Schuler et al., 2017).

**Figure 10:**
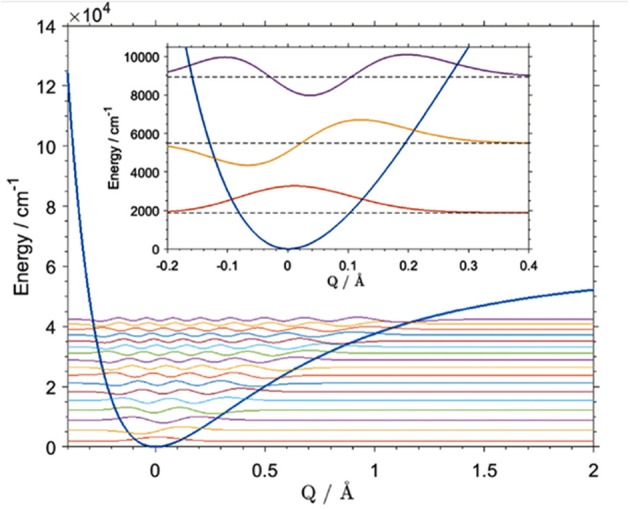
Vibrational potential and vibrational states [B3LYP/6-311G(d,p)] of the OH stretching mode of the main (equatorial-gauche) conformer of cyclohexanol. Reprinted with permission from Elsevier (Beć et al., 2018a).

A number of approaches to the described vibrational problem exist. The differences between them often results from various numerical methods being employed for solving the matrix differential equation in the eigenvalue problem (Schuler et al., 2017). These computations are often used in NIR physicochemical studies, where the precision of the calculation of a certain mode is essential. For example, Beć et al. recently showcased the potential of improving the quality of simulation of cyclohexanol in NIR (Figure 10; Beć et al., 2018a). In that case, the VPT2 method erroneously reproduced the 2νOH band, yielding a very high error on the wavenumber of the equatorial-*trans* form, the major conformer of the molecule. Consequently, a splitting of the 2νOH band appeared in simulation while it is absent in the experimental spectrum (Beć et al., 2018a). Dense grid-point probing [B3LYP/6-311G(d,p)+CPCM(CCl_4_)] of the vibrational potential along the OH stretching normal coordinate was carried out for the two leading conformers of cyclohexanol (Beć et al., 2018a). The generalized matrix Numerov method was used in solving the time-independent Schrödinger equation. By capturing the majority of anharmonicity, the vibrational levels were obtained with effectively absolute accuracy (<1 cm^−1^). This means that vibrational analysis was not a source of any meaningful error in itself. A reduction in the anharmonic vibrational analysis imprecision is essential, as it is sometimes a source of significant error (Schuler et al., 2017). However, the final inaccuracy vs. the experimental value is still affected by a number of other unavoidable factors, e.g., error on electronic energy or simplification of the molecular model such as single molecule calculations, implicit solvent models, etc. (Schuler et al., 2017). Luckily, that kind of error is frequently equal between similar molecules, e.g., the two cyclohexanol conformers. Effectively, high accuracy in relative sense in comparative studies is possible due to precise reproduction of the wavenumber differences existing between those systems. Beć et al. evidenced this circumstance well as they obtained an exact match of the splitting between 2νOH among the two conformers of cyclohexanol (calculated 30 cm^−1^, experimental 27 cm^−1^). By contrast, that value was predicted erroneously by VPT2 as 260 cm^−1^ (Beć et al., 2018a). Obviously, such quality of prediction requires large computing expense. The grid density may, however, be flexibly adjusted, which would allow for e.g., the affordable examination of larger molecules.

A highly accurate determination of vibrational states and transition intensities was performed e.g., in systematic examinations of subtle spectral variations due to certain substituents (Takahashi and Yabushita, 2013) or solvent effects (Futami et al., 2011, 2012; Gonjo et al., 2011) in NIR and MIR. For example, Gonjo et al. studied solvent effects in vibrational spectra of phenol and 2,6-dihalogenated derivatives (Gonjo et al., 2011). In this particular case, a high environmental sensitivity of the OH stretching mode delivered valuable spectral information; however, because of a high anharmonicity, this information is difficult for elucidation and accurate computations are essential. They examined fundamental, first-, second-, and third overtones by including broad spectral region, VIS, NIR, and MIR (15,600–2,500 cm^−1^). Such a wide aim necessarily required an accurate solution, particularly for the higher overtones. Therefore, the νOH vibrational potential was probed with a dense step (0.02 *q*_0_; −0.7 to 1.0 *q*_0_) using a relatively high level of electronic theory [B3LYP/6-311++G(3df,3pd)] for accurate energy evaluations; the vibrational problem was solved through Johnson's approach (Johnson, 1977). Implicit solvation by means of the Isodensity Polarizable Continuum Model (IPCM) allowed the reproduction of the experimental effects observable for phenol, 2,6-difluorophenol, 2,6-dichlorophenol, and 2,6-dibromophenol in different solvents (*n*-hexane, CCl_4_, CHCl_3_, CH_2_Cl_2_). Gonjo et al. concluded a “parity” in relative intensities over the sequences of transitions and they suggested that an intermolecular OH…Cl hydrogen bond between phenols and the solvent is responsible for that phenomenon (Gonjo et al., 2011). A similar problem was further investigated by Futami et al. for pyrrole (Futami et al., 2011). An accurate modeling of single-mode anharmonicity also enabled studies on the influence of the solvent's dielectric constant on X-H stretching mode in the solution phase, as reported by Futami et al. on the example of HF as a prototypic polar molecule (Futami et al., 2012). The computations [B3LYP/6-311++G(3df,3pd) and CCSD/aug-cc-pVQZ levels; IPCM solvent approximation] elucidated the potential and dipole moment function variations in response to the changing solvent's dielectric constant. The investigation of the solvent effect in NIR and IR was continued by Chen et al. (2014); in this case, C=O stretching vibrations in acetone and 2-hexanone were examined.

The formation of X-H…B hydrogen bonding induces significant changes to the potential curve, vibrational states and transition intensities of the stretching X-H vibration. Due to the high anharmonicity of this mode, accurate theoretical methods as discussed in this section are essential. This was demonstrated by Futami et al. on the example of the pyrrole, pyridine, and pyrrole–pyridine complexes (Futami et al., 2009). The experimental observation of non-bonded pyrrole reveals a well-resolved 2νNH peak at 6,856 cm^−1^; yet, the band is absent in the pyrrole–pyridine complex. A computational study explained that, in the complex the transition dipole moment is remarkably reduced for the first overtone mode of the hydrogen-bonded NH group (Futami et al., 2009). Continued further by Futami et al., other complexes featuring NH…π hydrogen bonding, e.g., pyrol-ethylene and pyrrole-acetylene systems, were evidenced to follow the above pattern (Futami et al., 2014).

### Toward Feasible Modeling of NIR Spectra of Complex Systems and Biomolecules

**Figure 11:**
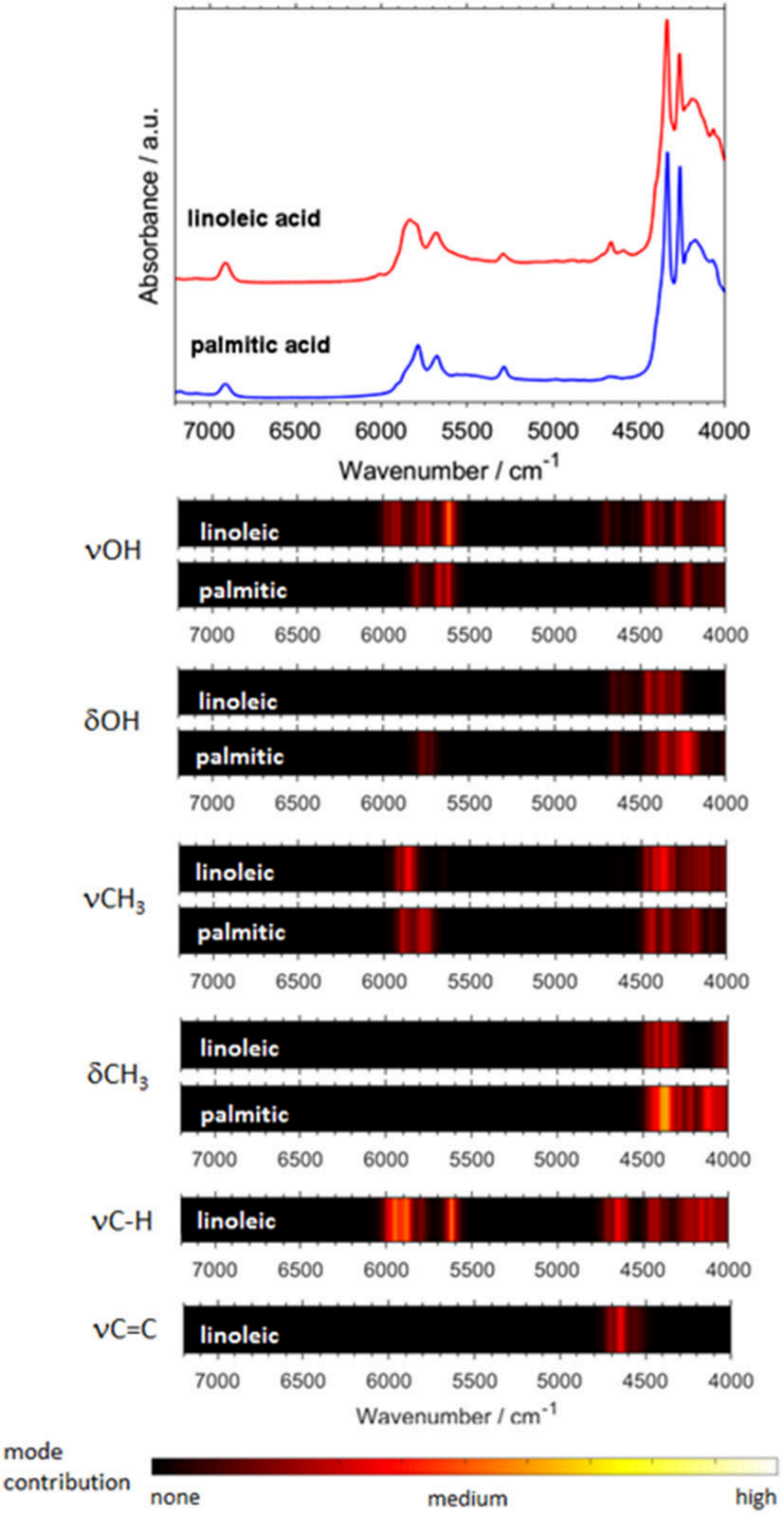
Generalized contributions into NIR spectra of the selected types of modes involved in the binary combinations as uncovered by quantum mechanical spectra simulation of linoleic and palmitic acid. (Reprinted with permission from Grabska et al. (2018). Copyright (2018) American Chemical Society).

Biomolecules feature a largely increasing importance in current spectroscopy in response to the strong stimulus for boundary-crossing research e.g., with focus on medicinal applications Jue and Masuda (2013). This also holds for NIRS, which is useful for investigations of biomaterial owing to its capability of studying moist samples. As it was well-explained throughout this review so far, biomolecules are complex systems which prove to be a particular challenge for theoretical NIRS. It is, therefore, of high importance to advance toward efficient NIR simulations of biomolecules. Because of this, recently matured VPT2 routines (DVPT2/GVPT2) seem to be the most promising due to their favorable cost-to-accuracy ratio (see section Fundamentals of Theoretical NIRS). Among the major kinds of biomolecules, long-chain fatty acids (LCFAs) have the advantage of forming better-defined structures, due to the tendency of carboxyl groups to form cyclic dimers strongly stabilized through dual hydrogen-bond (see section Investigations of Intermolecular Interactions). LCFAs feature protracted aliphatic chains (13–22 carbons); those most commonly appearing (e.g., oleic acid or palmitic acid) have chains with 15 to 22 carbons. They exist in all kinds of biological matter, either as the constituents of lipids or as free fatty acids (FFAs). They are the second energy source of the animal body and are also part of the chemical composition of several vegetable oils (Zielinska, 2014). They also suit a wide range of industrial applications, thus remaining the focus of NIRS in pharmaceutical (Ahmad, 2017), cosmetics (Białek et al., 2016), and food industries (Wood et al., 2003). For these reasons, the relevancy of these biomolecules in the context of NIRS is very high. Hence, LCFA molecules suited as perfect objects for the breakthrough study as reported recently by Grabska et al. (2018). A selection of six compounds, saturated (palmitic, stearic, arachidic) and unsaturated (linolenic, linoleic, oleic) acids, was investigated by experimental/theoretical NIRS. These examinations were aimed at providing an in-depth explanation of the spectral origins, their relationships to the chemical structure, in particular the impact of alkyl chain's saturation, as well as deeper insights into anharmonicity of the most important vibrational modes in these molecules (Grabska et al., 2018). Two separate computational approaches were used in this study. The first one, DVPT2, was an effective way to reproduce the entire NIR spectra and proved that the cost-effectiveness of the method is adequate even for biomolecular studies (Ozaki et al., 2017). The accurate reproduction of the NIR lineshapes proved that the approximation of inter-modal anharmonicity, i.e., coupling between vibrational modes, in DVPT2 retains adequate quality even for the molecular systems with an overwhelming number of such couplings; the number of binary combinations surpassed 66,000 for the dimeric molecules of LCFAs (Beć and Grabska, 2018; Grabska et al., 2018). This fact also reflects the exponentially increasing complexity of the NIR spectra with the size of a molecule. The resulting combination band overlay creates substantial difficulty in elucidating certain spectra-forming factors and comparing specific features, e.g., the impact of the saturation of LCFAs. For this reason, Grabska et al. presented a clearer and more accessible illustration of the spectral contributions in the form of heatmap (Figure 11). These maps reflect the level-of-influence of selected modes of interest, arbitrarily grouped in such a way that the important factors could be easily assessed. The quality of simulation allowed for unambiguous assignment of all NIR bands and also to differentiate clearly between saturated and unsaturated LCFAs. Further progress in this field is strongly promoted as it finds immediate application in NIR biospectroscopy and imaging (Grabska et al., 2017c,d; Puangchit et al., 2017).

### Theoretical NIRS in Aid of Analytical Applications

Bio-significant molecules remain near the center of attention of applied NIRS. Qualitative and quantitative analysis of natural products (e.g., raw biomaterials, intermediate and final products) is a strongly developing field of applications (Huck, 2014, 2016a); this fact focuses attention on biomolecules. NIRS in hyphenation with chemometrics (multivariate analysis; MVA) uses rich chemical information entangled in the NIR spectrum of complex samples and correlates it with the chosen property of sample (Ozaki et al., 2006). Despite practical effectiveness, the procedure is performed in black-box from the point of view of physical chemistry. Molecular and vibrational background phenomena remain transparent in this routine. It has been a necessity for analytical NIRS to evolve despite such hindrance. The concept of incorporating chemical band assignments into chemometrics appeared in literature (Westad et al., 2008) with the aim to improve the analytical performance of NIRS. However, this idea has not been truly employed further. The lack of readily available high-resolution deconvolution of the spectra, which would additionally offer an ability to assign the resolved contributions was the major problem here. QM simulation of NIR spectra bears a strong potential to finally reactivate this extremely promising line of research. Recent time has seen attempts to adopt theoretical NIRS for the benefit of analytical studies (Schmutzler et al., 2013; Lutz et al., 2014a; Kirchler et al., 2017a; Kirchler et al., 2017b).

The first of such an example appeared in 2014 as Schmutzler et al. reported their design of an analytical pathway which employs NIRS/MVA for the effective quality control of apples (Schmutzler et al., 2013). This contribution had a substantial impact on the quality control of food/agricultural products, as it proposed entirely automatic, non-destructive NIRS instrumentation for the analysis of apples. It utilized the principle of surface scanning in order to average the sample's surface inhomogeneity and a fiber probe for the convenient arrangement of the analytical instrumentation. From the point of view of the present review, it should be emphasized that this work also employed QM spectra simulation in the procedure, resulting in a first attempt of creating a computer-aided NIRS analytical spectroscopy. In this study, the point of interest was put on malic acid, a dicarboxylic acid existing on apple surface, which is a highly informative marker of the fruit's general condition (Schmutzler et al., 2013).

**Figure 12:**
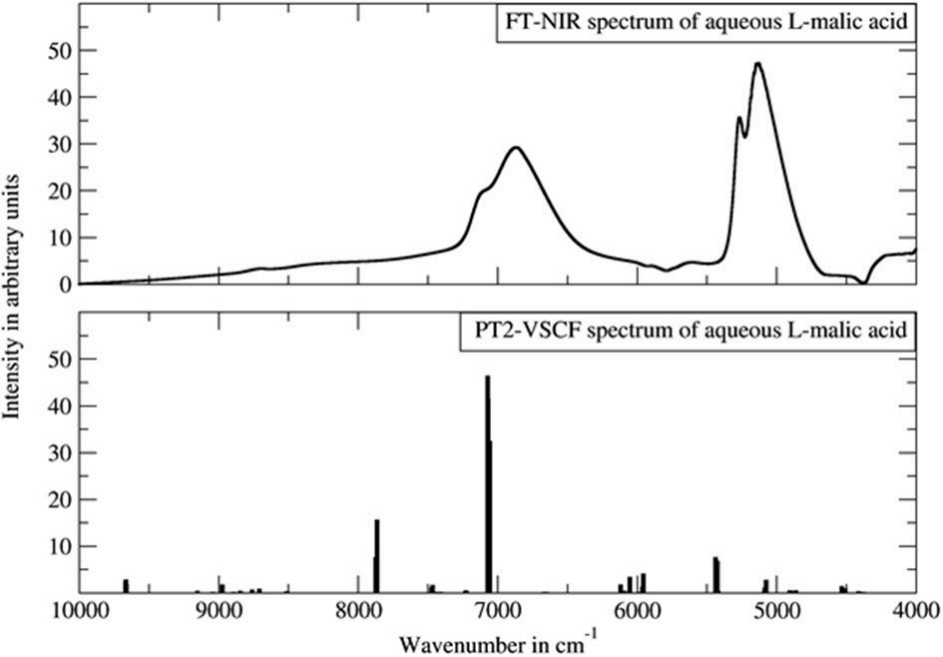
The experimental FT-NIR spectrum of aqueous malic acid in comparison with the PT2-VSCF derived line spectrum. Reprinted from Schmutzler et al. (2013). Reprinted with permission from Nova Science Publishers, Inc.].

For the anharmonic vibrational analysis of malic acid, Schmutzler et al. used PT2-VSCF, a second-order perturbation corrected VSCF scheme, which features an improved quality of prediction of inter-modal anharmonicity (Schmutzler et al., 2013). The electronic structure was determined at the Møller-Plesset MP2 level of theory with a 6-31G(d,p) basis set; an implicit approximation of aqueous solution through CPCM solvation model of water was included. L- and D- chiral isomers of malic underwent anharmonic treatment and theoretical bands up to three quanta, second overtones and ternary combinations, were obtained. Simulated bands compared to the experimental NIR spectrum are presented in Figure 12. The experimental features were reflected in the simulation qualitatively, correctly yielding comprehensive band assignments. According to the authors, the assumed simplifications i.e., quartic force field approximation, relatively simple basis set, implicit solvation model, could decrease the accuracy of their simulation. They also concluded that the PT2-VSCF approach involves only moderate computational efficiency which is further lowered by unfavorable scaling with molecule size. As they pointed out, this considerably hinders the feasibility of studying larger molecules using similar methods (Schmutzler et al., 2013). Notwithstanding this, simpler molecules such as malic acid may successfully undergo PT2-VSCF treatment in aid of analytical NIRS. Accordingly, Lutz et al. applied a similar computations scheme in their development of miniaturized NIRS for gasoline content analysis and quantification (Lutz et al., 2014a). Several kinds of compounds were needed to be considered, in order to represent the chemical residents in gasoline. Consequently, they examined ethanol (oxygenated fuel additive), *n*-octane (linear alkane representative), toluene (aromatic and branched/alkyl-substituted aromatic hydrocarbons) and ethyl *tert*-butyl ether (ethers and branched/alkyl-substituted aliphatic hydrocarbons and also oxygenated fuel additive). These systems retain relative simplicity and are suitable for PT2-VSCF anharmonic analysis without an apparent need for simplifications. This even allowed for the employment of the post-Hartree-Fock method for the determination of electronic energy, as the MP2 method together with TZVP basis set were used. Thus, the level of electronic theory was considerably higher than in the study of malic acid. In this case, Lutz et al. succeeded in inserting QM simulation into their analytical study, and the benefits included comprehensive band assignments, vastly improving the qualitative discrimination of the gasoline residents (Lutz et al., 2014a).

**Figure 13:**
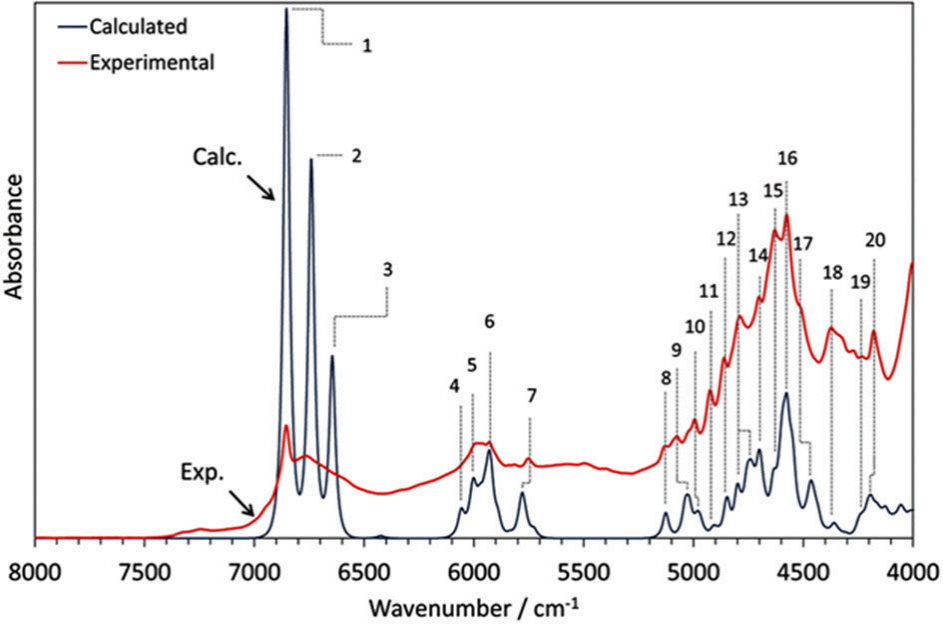
The experimental (powder) and theoretical NIR spectrum of rosmarinic acid obtained in anharmonic GVPT2//DFT-B3LYP/N07D simulation (Kirchler et al., 2017b). Reproduced by permission of The Royal Society of Chemistry.

Phytoanalysis is one of the fields in which analytical NIRS becomes the tool-of-choice in qualitative and quantitative analysis (Huck, 2014, 2016a). The phytopharmaceutical industry requires highly robust methods of analysis as natural products are complex and susceptible to content variation. Hence, an independent insight from the NIR simulation brings in a substantial value, e.g., by allowing the qualitative evaluation of analyzed data or the comprehension of chemical factors influencing chemometric analysis. Recently, Kirchler et al. showcased the potential of combined theoretical/analytical NIRS in two of their subsequent feasibility studies on the analytical performances of miniaturized NIRS in quantifying rosmarinic acid (RA) in *Rosmarini folium* (Kirchler et al., 2017a,b). In various traditional plant medicines, RA is the primary active compound with therapeutic and antioxidant properties. As a relatively complex molecule (42 atoms; 188 electrons), it is somewhat expensive to obtain the anharmonic force field. For a similar reason, the resulting NIR spectrum involves numerous convoluted bands. This stems from a high number of binary combinations (7,140 in total), and naturally, a similarly substantial amount of intermodal couplings required to simulate the NIR spectrum of RA. To achieve the best cost/accuracy balance, the DVPT2 approach at the B3LYP/N07D level of electronic theory were employed for this task (Figure 13). This selection offers good efficiency without any essential accuracy compromise. As showcased in the case of RA, it delivered a qualitatively correct result and may be useful in simulating large molecules. A simplified treatment of the RA molecule in a vacuum did not reduce the quality of simulation exceedingly; manifestations of intermolecular interactions e.g., 2νOH band broadenings could be easily identified in the experimental spectrum with the availability of a clear image resolved from QM simulation. Additionally, the baseline elevation effects could also be understood, much like the effects observed previously for carboxylic acids (Grabska et al., 2017c,d). These accomplishments yielded full comprehension of the NIR spectrum of RA, leading to detailed band assignments (Figure 13 and Table 2). These findings could then be used by Kirchler et al. for gaining a better understanding of the variability of PLS regression coefficients vectors assembled for the data originating from different NIR spectrometers with particular attention paid to miniaturized devices (Kirchler et al., 2017b). Different instruments yield different calibration curves, and different wavenumber regions manifest varying levels of influence in the regression. Kirchler et al. presented a state-of-the-art multi-method approach combining QM simulations, chemometrics (involving wavenumber discriminant methods, e.g., Moving-Window PLSR, MW-PLSR), and advanced methods of spectral analysis (Two Dimensional Correlation, 2D-COS; in particular, heterocorrelation elucidating spectral differences due to instrumental factors), indicating how modern analytical NIRS may be evolving in the foreseeable future (Kirchler et al., 2017b).

**Table 2:** Band assignments in NIR spectrum of rosmarinic acid, based on GVPT2//DFT-B3LYP/N07D calculation.

	**Wavenumber/cm**^**−1**^		**Major contributions**
	**Exp**.	**Calc**.	
1	6854.9	6,853	2νOH (ar)
2	6767.2	6,741	2νOH (ar)
3	~6680	6,645	2νOH (carboxyl)
4	~6044	6,056	2νCH (ar, aliph, in-phase)
5	5986.5	6,001	2νCH (ar, aliph, opp.-phase); 2νCH (ar)
6	5929.7	5,930	2νCH (ar); 2νCH (ar)
7	5752.5	5,780	[ν_as_CH_2_, νCH (ar)] + [ν_as_ CH_2_, νCH (ar)];
8	5128.0	5,126	[ν C = O, δ_ip_OH (carboxyl)] + [νOH (carboxyl)]
9	5075.8	5,027	[δ_ring_, δ_ip_OH (ar)] + [νOH (ar, para-)]; [δ_ring_, δ_ip_OH (ar)] + [νOH (ar, para-)]; [δ_ring_] + [νOH (ar, para-)]
10	4994.9	4,980	[δ_ring_, δ_ip_OH (ar)] + [νOH (ar, meta-)]; [δ_ring_, δ_ip_OH (ar)] + [νOH (ar, meta-)]
11	4923.8	4,906	[δ_ring_, δ_ip_OH (ar)] + [νOH (ar, para-)]; [δ_ring_, δ_ip_OH (ar)] + [νOH (ar, para-)]
12	4860.0	4,847	[δ_ring_, δ_ip_OH (ar)] + [νOH (ar, meta-)]; [δ_ring_, δ_ip_OH (ar)] + [νOH (ar, meta-)]
13	4788.3	4,798	[νCC] + [νOH (ar, para-)]; [νCC] + [νOH (ar, para-)]; [νCC] + [νOH (ar, meta-)]; [δCCH (carboxyl)] + [νOH (carboxyl)]; [δCH (ar), δ_ip_OH (ar)] + [νOH (ar, para-)]; [δCH (ar), δ_ip_OH (ar)] + [νOH (ar, para-)]
14	4701.0	4,701	[δCH (aliph)] + [νOH (ar, meta-)]; [δCH (ar), δ_ip_OH (ar)] + [νOH (ar, meta-)]
15	4629.4	4,632	[δCH (ar), δ_ring_, δ_ip_OH (ar)] + [νOH (ar, meta-)]; [δCH (ar), δ_ring_, δ_ip_OH (ar)] + [νOH (ar, para-)]; [δ_ip_OH (ar), δCH (ar), δ_ring_] + [νOH (ar, para-)]
16	4575.7	4,757	[δCH (ar), δ_ring_, δ_ip_OH (ar)] + [νOH (ar, meta-)]; [δ_ip_OH (ar), δCH (ar), δ_ring_] + [νOH (ar, meta-)]
17	~4,508	4,465	[δ_ring_, δ_ip_OH (ar)] + [νCH (ar)]; [δ_ring_] + [νCH (ar)]; [δ_ring_, δ_ip_OH (ar)] + [νCH (ar)]; [νC-O (carboxyl), δ_ip_OH (carboxyl)] + [νOH (carboxyl)]; [δ_ring_] + [νCH (ar)]; [δ_ring_, δ_ip_OH (ar)] + [νCH (ar)]
18	4372.3	4,360	[δ_ring_] + [νCH (ar)]; [δ_ring_] + [νCH (ar)]
19	4233.3	4,237	[δ_sciss_CH_2_] + [ν_as_CH_2_, νCH (ar)]; [δ_sciss_CH_2_] + [ν_as_CH_2_, νCH (ar)]
20	4179.4	4,194	[δCH (aliph)] + [νCH (ar, aliph, opp.-phase)]; [δ_sciss_ CH_2_] + [ν_s_CH_2_]; [δCH (aliph)] + [νCH (ar, aliph, in-phase)]

*Kirchler et al. (2017b)-Reproduced by permission of The Royal Society of Chemistry*.

**Figure 14:**
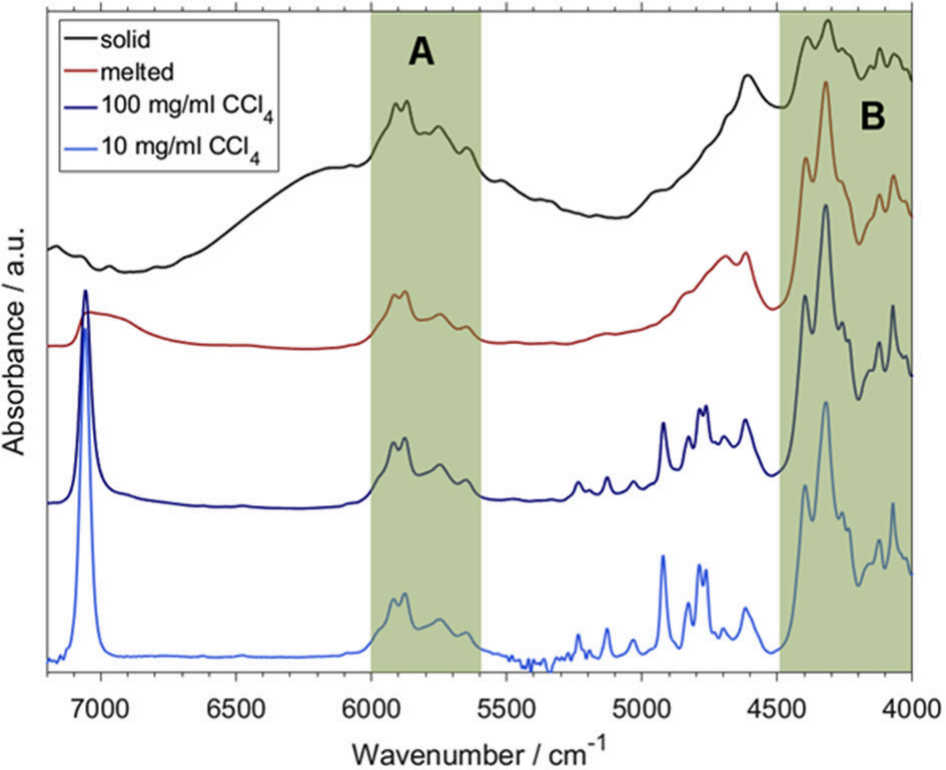
A set of the experimental NIR spectra of thymol; solid state and melted (neat liquid, 333 K) as well as diluted in CCl_4_ (100 and 10 mg mL^−1^ CCl_4_). Highlighted are the wavenumber regions qualitatively independent of sample phase and concentration; **(A)** 6,000–5,600 cm^−1^; **(B)** 4,490–4,000 cm^−1^ (Reprinted with permission from Beć et al., 2018b).

**Figure 15:**
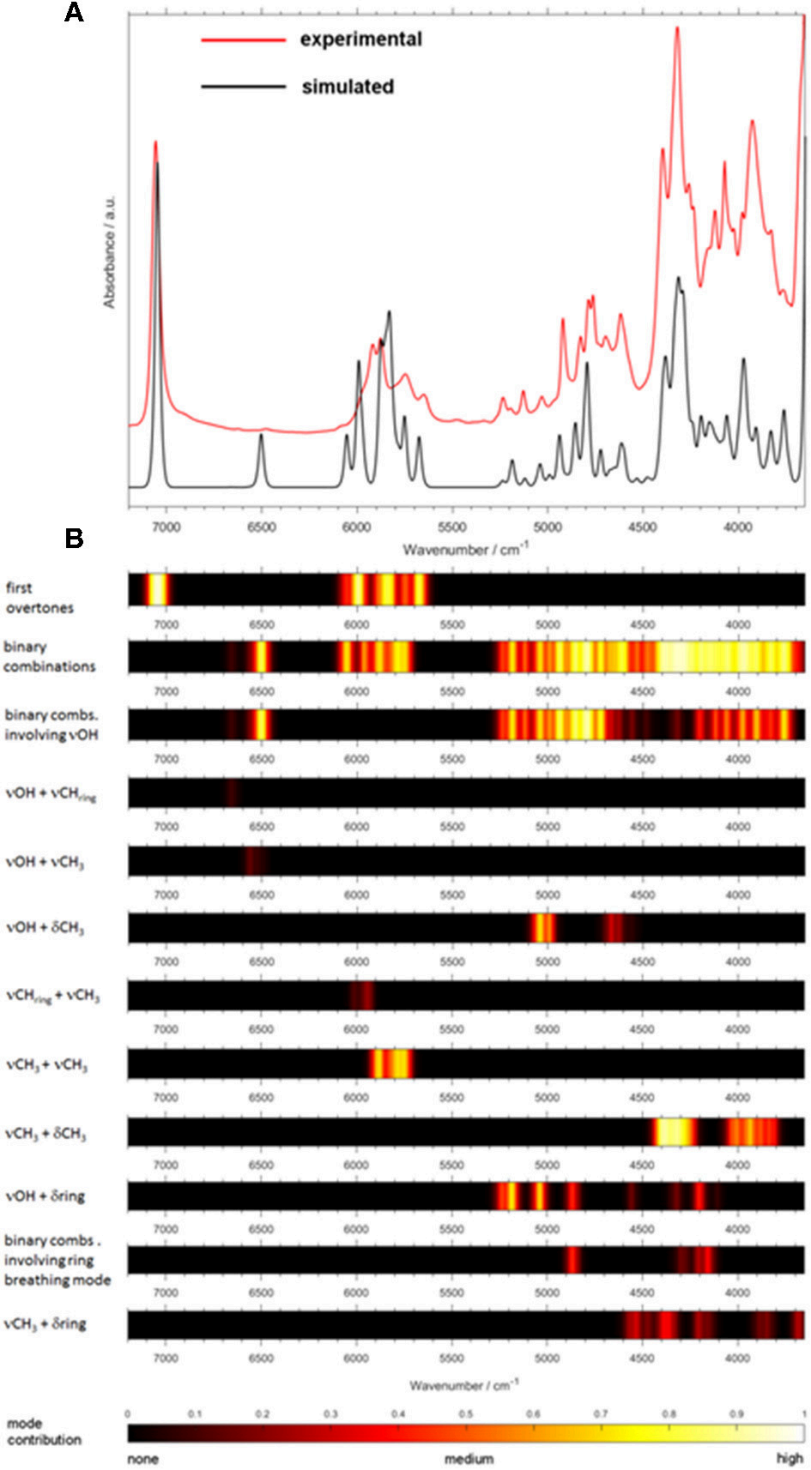
The analysis of mode contribution into NIR spectrum of thymol (solution; 100 mg mL^−−1^ CCl_4_) based on the simulated data (DVPT2//DFT-B3LYP/SNST+CPCM). **(A)** Experimental and simulated outlines. **(B)** Contributions of selected modes as described on the figure (Reprinted with permission from Beć et al., 2018b).

A more recent study confirmed the feasibility of the concept outlined above and went a step further in exploring how QM spectra simulations can be used to obtain new insight and physicochemical interpretation of the predictive models benefitting analytical applications (Beć et al., 2018b). Beć et al. investigated NIR properties of thymol with a handful of highly informative findings which would be otherwise unobtainable without accurate NIR simulation. Thymol is a phenolic constituent commonly found in a number of herbal plants, e.g., in a traditional plant medicine Thymi herba. Thymol strongly contributes to the general therapeutic properties of these herbs; e.g., by its anti-oxidant, anti-inflammatory, antiseptic (antifungal and antibacterial), antispasmodic, and immunomodulatory properties. Beyond its pharmaceutical significance, thymol is also an interesting molecule for spectroscopic investigations because of its vibrational features. The side groups attached to an aromatic ring may provide plentiful information on the spectra-structure correlations. An OH group commonly manifests a strong tendency to interact with the chemical neighborhood, e.g., to form a hydrogen-bond network. Due to this fact, and also because of the high anharmonicity of its vibrations, the existence of an OH group is a major spectrum-forming factor in NIR. For these reasons, the OH group has frequently been in the center of attention of NIR physicochemical as found in the literature (Czarnecki et al., 2015). The examination by Beć et al. elucidated a pattern of spectral variability following the sample state (solid and melted, neat liquid) and concentration (neat liquid and diluted in an inert solvent; CCl_4_). Certain spectral regions in NIR manifested strong insensitivity to those properties of the sample (Figure 14; regions highlighted as A and B), while some others were observed to be clearly affected (Figure 14; all bands outside regions A and B). By QM simulations, the modes which stand behind that consecutive pattern could be fully identified (Figure 15). It was then observed that there exists a clear division between the highly relevant factors from the point of view of NIRS. Vibrations which are the most essential in shaping the NIR spectrum, and also sensitive to the changes in the sample property, were found to be consecutively discriminated by the PLS regression while quantifying the thymol content in a natural sample. Upon confronting these influential wavenumbers with the major features in the PLSR coefficients vector, it was found that the entirety of the spectral features identified as decisive in the PLSR model (5,860, 5,760; falling into the region A; Figure 14 and 4,476, 4,418, 4,392, 4,368, 4,220, 4,128, and 4,092; cm^−1^; belonging in the region B; Figure 14) originate from two insensitive to the concentration and sample state NIR sub-regions of thymol. These “invariant” regions contain CH_3_ and aromatic CH bands. Surprisingly, the νOH mode, which is otherwise the most influential spectrum-forming factor (Figure 15), was largely omitted in the chemometric model. It was suggested that the sensitivity of the νOH mode to intermolecular interactions, manifested in NIR through significant band broadening, may be one of the responsible factors (Beć et al., 2018b). That promising study demonstrated well the need to further explore through a systematic investigation the underdeveloped area which appears at the connection between physical chemistry, theoretical NIRS and applied spectroscopy.

### Other Applications, Arising Possibilities, and Remaining Challenges

As explored so far in the present review, the advent of feasible NIR spectra simulations has brought substantial gains to physical and analytical chemistry, both in basic and applied studies. The benefits for biomedical applications, e.g., NIR hyperspectral imaging (mapping) of biosamples could be evidenced as well (e.g., Huck, 2016a; Ozaki et al., 2017; Türker-Kaya and Huck, 2017). Deep penetration of the sample by NIR light, a consequence of the typically low absorptivity in this spectral region (see section Near-Infrared Spectroscopy. The Tale of an Ugly Duckling), forms a perfectly synergistic effect with the other values of NIRS (e.g., non-invasive character, low cost factor, wide applicability, superior time-to-result ratio), creating a strong stimulus for further development at the foundations of this technique (He et al., 2018; Wong et al., 2019). A question may arise on how in the nearest future theoretical and computational methods may become useful in these developments.

Several topics of the highest importance for spectral imaging presently remain a subject of intensive research, with the ultimate goal of introducing a feasible multi-modal imaging technique (He et al., 2018). A good example is the recent development of NIR radiation sources (He et al., 2015; Sun et al., 2017; Wong et al., 2019), with aim of being used in NIR fluorescence imaging, as thoroughly reviewed by He et al. (2018). The development studies for novel sources utilizing small-molecule fluorophores by Sun et al. (2017), high-efficiency NIR emitting materials coming from Wong et al. (2019), or ultralow-intensity NIR radiation source for drug delivery using upconverting particles by He et al. (2015), could potentially see substantial gains from readily available theoretical and computational approaches. The new radiation sources which yield their superior capabilities from novel applications of bioinorganic chemistry, e.g., heavy transition metal and lanthanide complexes, could potentially benefit from computational simulations of the vibrational and electronic properties of such materials. At present, this remains a challenge, due to the complex electronic structure of transition metal atoms. Anharmonic simulations of vibrational spectra of such materials are prohibitively expensive; the computational expense associated with solving the vibrational problem in anharmonic approximation of advanced materials requires well-thought simplifications applied at several potential levels, ranging from the vibrational analysis itself (e.g., through reducing the grid density for total energy evaluations; Lutz et al., 2014c) to ingenious approaches to the electronic structure itself (e.g., Messner et al., 2014; Lutz et al., 2015). Vibrational spectra simulations of the metaloorganic complexes involving lighter metal ions can be found in recent literature.

As a good example, Lutz et al. have succeeded in the accurate reproduction of vibrational properties of the bioinorganic complex, trans-bis(glycinato)copper(II) (*cis* and *trans* isomers), in an aqueous solution (Lutz et al., 2013). They conducted quantum mechanical charge field molecular dynamics (QMCF-MD) studies, reporting the first QM simulations of organometallic complexes by this method. In hyphenation with experimental MIR spectroscopic data, they yielded accurate structural details of the investigated isomers as well as novel dynamic data, which has successfully been confirmed and extended by subsequent mid-infrared measurements. Although still being limited to a scaled harmonic approximation, according to Lutz et al. (2013), the spectroscopic results, critically assessed by adjacent multivariate data analysis (chemometrics), indicated an isomeric stability at ambient conditions, vanishing at elevated temperatures. Chemical systems containing metal ion significantly increase the computational complexity of the simulation procedure. Often, while maintaining careful error control, simplifications in the determination of the electronic structure may bring substantial gains in this regard. As recently demonstrated by Messner et al. in their investigation of the hydration of immobilized Fe(III), complexes of Fe(III) with methyl substituted iminodiacetate ([Fe(MSIDA)(H_2_O)_3_]+), as well as with methyl substituted nitrilotriacetate ([Fe-(MSNTA)(H_2_O)_2_]) in aqueous solutions (Messner et al., 2014) by QMCF-MD, ingenious balancing between the cost-accuracy of the applied electronic method brings notable advantages, e.g., allowing to expand the parameters of molecular dynamics in return. By choosing a relatively fundamental Hartree-Fock (HF) approach, even over a much more robust MP2 method, Messner et al. accomplished essential progress in our understanding of the hydration structure and dynamics of metaloorganic complexes. Their effort has also been aimed at vibrational properties, while MIR spectroscopic data has been used as the reference. The understanding of the dynamics of metal cations and metaloorganic complexes in aqueous solution progressed further with ongoing studies continued within the same research group, e.g., as reported by Tirler et al. (2015) who once again applied, with high success, the quantum dynamic QMCF-MD simulation for the exploration of the stability of aqueous hexacyanoferrate(II) ion, in isolation as well as in the presence of potassium counterions. Further progress has been achieved in understanding the structure and dynamics of solvated metaloorganic complexes, when Tirler and Hofer investigated [MgEDTA]2– and [CaEDTA]2– systems (Tirler and Hofer, 2015). Furthermore, even more sophisticated systems, such as the aqueous 18-crown-6 (18C6) and strontium(II)-18-crown-6 (18C6–Sr) have been proven (Canaval et al., 2015) to be applicable to QMCF-MD treatment.

So far, simulation studies have repeatedly been evidenced to be of high value to metaloorganic chemistry, by delivering unique and highly desired information on the structure, vibrational features, dynamics, solvation, interactions, and stability of these important constituents of novel materials. It may be anticipated that these accomplishments will result in further progress in the computer-aided material design, which could find immediate application in the development of novel chromophores and enhancing NIR spectral imaging. On the other hand, the evolution toward anharmonic approximation may bring feasible studies of the properties of such materials in the NIR region. Currently, this remains hindered by the computational complexity introduced through anharmonic approximation, which indirectly also makes it difficult to fully incorporate a number of other effects, (e.g., relativistic effects Kondo et al., 2018; Madhu Trivikram et al., 2018), spin-orbit interaction (Gans et al., 2013), or vibronic coupling (Bloino et al., 2016). On the other hand, the exploration of the possible ways to increase the affordability of the relevant computational approaches (e.g., Lutz et al., 2014c, 2015; Messner et al., 2014) is ongoing, as explained above, and substantial advancements may be anticipated to occur in the near future.

## Final Remarks and Future Prospects

Analytical NIRS relies on correlating the spectral variability with sample properties. The tools used for this purpose do not provide any understanding of these correlations, e.g., the parameters of the chemometric models have no immediately available physical interpretation. This creates a serious hindrance for applied NIRS from a conceptual, but also from a practical, point of view. For example, it has been shown that the structure of the PLS regression coefficients vector changes, e.g., between different spectrometers. Sensitivity to a multitude of different factors makes it difficult to elucidate the vibrational background of the analyzed spectral variability and the role of anharmonic effects. Theoretical spectroscopy offers substantial aid in answering the principle questions, which would be beneficial for both basic NIRS in the physicochemical context and in applied analytical spectroscopy.

Theoretical NIRS currently stands in a unique spot, where its usefulness to applied spectroscopy is far superior than the analogous relationships present in other kinds of vibrational spectroscopy. Nowadays, theoretical NIRS is still at a relatively early stage of development. It is an emerging field, which only recently became more explored as the associated computational complexity had long been prohibitive. It was the current decade which witnessed advances in anharmonic theories, aided by ever-growing computer technology, which has enabled the feasible theoretical NIRS in connection with applied spectroscopy. As the number of studies in this area develops, the link between analytical and theoretical NIRS is being further strengthened; a clear trend in this evolution path is marking the anticipated further advancement. Focus of applied spectroscopy is on complex samples in which molecules remain under constant influence of the chemical neighborhood througha variety of intermolecular interactions, e.g., as in the discussed case of malic acid-water complex (Schmutzler et al., 2013). One should anticipate that in the near future research will be oriented to this direction, with more complex and interacting molecular systems, large biological systems and direct applications of theoretical NIRS in analytical routines. An even more coherent growth of theoretical near-infrared spectroscopy in close connection to analytical applications may be envisioned.

## Author Contributions

KB designed the article and wrote the manuscript. CH co-designed the general outline of the article. Both authors discussed the details of the review.

### Conflict of Interest Statement

The authors declare that the research was conducted in the absence of any commercial or financial relationships that could be construed as a potential conflict of interest.

## Footnotes

^1^This includes terahertz (THz) spectroscopy; despite differences in the instrumentation, FIR and THz spectroscopy both elucidate the same information from the analyzed sample (Mantsch and Naumann, 2010).

^2^(http://www1.lsbu.ac.uk/water/water_vibrational_spectrum.html#k) under Creative Commons license. Attribution-NonCommercial-NoDerivs 2.0 UK: England & Wales (CC BY-NC-ND 2.0 UK).
